# Discovery of Novel Piperidinyl-Based Benzoxazole Derivatives as Anticancer Agents Targeting VEGFR-2 and c-Met Kinases

**DOI:** 10.3390/ph18121875

**Published:** 2025-12-09

**Authors:** Wagdy M. Eldehna, Zainab M. Elsayed, Mohamed R. Elnagar, Ahmed H. El-Said, Taghreed A. Majrashi, Ahmed T. Negmeldin, Abdulrahman M. Saleh, Ranza Elrayess, Khaled A. Elnahriry, Zhi-Long Chen, Mohamed Elagawany, Haytham O. Tawfik

**Affiliations:** 1 Department of Pharmaceutical Chemistry, Faculty of Pharmacy, Kafrelsheikh University, Kafr El-Shaikh 33516, Egypt; wagdy2000@gmail.com; 2 Scientific Research and Innovation Support Unit, Faculty of Pharmacy, Kafrelsheikh University, Kafr El-Shaikh 33516, Egypt; zienabmohammed9@gmail.com; 3 Department of Pharmacology and Toxicology, Faculty of Pharmacy, Al-Azhar University, Cairo 11823, Egypt; mohamed.r.elnagar@azhar.edu.eg; 4 Department of Pharmacology, College of Pharmacy, The Islamic University, Najaf 54001, Iraq; 5 Department of Pharmaceutical Chemistry, Faculty of Pharmacy, Delta University for Science and Technology, International Coastal Road, Gamasa 11152, Egypt; ahmedhamdysaid@yahoo.com; 6 Department of Pharmacognosy, College of Pharmacy, King Khalid University, Asir 62521, Saudi Arabia; tamajrashi@kku.edu.sa; 7 Department of Pharmaceutical Sciences, College of Pharmacy and Thumbay Research Institute for Precision Medicine, Gulf Medical University, Ajman 4184, United Arab Emirates; 8 Department of Pharmaceutical Organic Chemistry, Faculty of Pharmacy, Cairo University, Cairo 12613, Egypt; 9 Department of Pharmaceutical Chemistry, Faculty of Pharmacy, Cairo University, Kasr El-Aini Street, Cairo 11562, Egypt; abdo.saleh240@azhar.edu.eg; 10 Pharmaceutical Organic Chemistry Department, Faculty of Pharmacy, Suez Canal University, Ismailia 41522, Egypt; ranza.el-rayes@pharm.suez.edu.eg; 11 Pharmaceutical Organic Chemistry Department, College of Pharmacy, Al-Ayen Iraqi University (AUIQ), An Nasiriyah 64001, Iraq; 12 Department of Pharmaceutical Sciences, College of Pharmacy, Alfaisal University, Riyadh 11533, Saudi Arabia; kelnahriry@alfaisal.edu; 13 Department of Pharmaceutical Science and Technology, Donghua University, Shanghai 201620, China; zhlchen@dhu.edu.cn; 14 Department of Pharmaceutical Chemistry, Faculty of Pharmacy, Damanhour University, Damanhour 22516, Egypt; elagawany@pharm.dmu.edu.eg; 15 Department of Pharmaceutical Chemistry, Faculty of Pharmacy, Tanta University, Tanta 31527, Egypt

**Keywords:** piperidinyl benzo[*d*]oxazole, c-Met, VEGFR-2, RT-PCR, apoptosis, in silico

## Abstract

**Background/Objectives**: A promising anticancer strategy is the simultaneous inhibition of the receptor tyrosine kinases VEGFR-2 and c-Met, which are essential for tumor angiogenesis, growth, and metastasis. In this study, a novel series of piperidinyl-based benzoxazole derivatives was designed and synthesized as potential dual VEGFR-2/c-Met inhibitors. **Methods**: The kinase inhibitory potential of the derivatives was evaluated in comparison to reference inhibitors, Sorafenib (VEGFR-2 inhibitor) and Staurosporine (c-Met inhibitor). Cytotoxicity was assessed across breast, prostate (PC-3), and lung (A549) cancer cell lines. Mechanistic studies included cell-cycle analysis, apoptosis assays, gene expression profiling of apoptosis-related markers, and molecular docking within the ATP-binding pockets of both kinases. **Results**: Compounds **5a**, **5g**, **5h**, **11a**, and **11b** showed strong inhibition of both kinases (IC_50_ = 0.145–0.970 μM for VEGFR-2 and 0.181–1.885 μM for c-Met). Selective cytotoxicity was observed against breast cancer cells, with compound **11b** (*p*-fluorophenyl derivative) exhibiting high selectivity toward MCF-7 over normal breast cells (MCF-10A) and potency comparable to or exceeding Sorafenib. Mechanistically, **11b** induced G_2_/M cell-cycle arrest and apoptosis (total apoptosis = 48.34%), accompanied by upregulation of p53, BAX, and caspase-9 and downregulation of Bcl-2. Molecular docking confirmed stable binding within the ATP-binding sites of both kinases. **Conclusions**: Compound **11b** was established as a novel, selective, dual VEGFR-2/c-Met inhibitor with strong potential for targeted breast cancer therapy.

## 1. Introduction

The ability of tumor cells to adapt to harsh microenvironments, unchecked cell proliferation, and genetic instability makes cancer one of the world’s leading causes of morbidity and mortality [1]. Hypoxia is one of the main characteristics of solid tumors and is brought on by aberrant vasculature, altered metabolism, and unchecked development. The hypoxia-inducible factor-1α (HIF-1α) pathway is the primary mediator of adaptive signaling systems that tumor cells activate in response to hypoxic stress [2]. By controlling the expression of several angiogenic mediators, including the vascular endothelial growth factor receptor-2 (VEGFR-2), this transcription factor promotes angiogenesis, tumor growth, and metastasis, all of which contribute to worsening patient outcomes [3].

**Figure 1:**
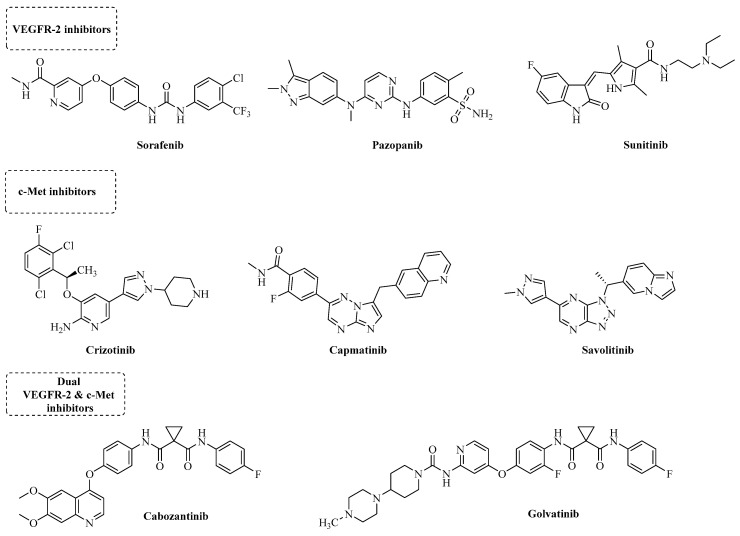
Structure of some reported active agents towards VEGFR-2 and c-Met.

A receptor tyrosine kinase called VEGFR-2 is crucial for angiogenesis and is frequently overexpressed in various cancers. Its activation sets off subsequent signaling cascades that support the migration, proliferation, and survival of endothelial cells [4]. Numerous VEGFR-2 inhibitors [5], including axitinib, sunitinib, pazopanib, and sorafenib, have demonstrated notable therapeutic activity and have received clinical approval (Figure 1). However, because tumors quickly gain resistance, the therapeutic effects of VEGFR-2 inhibition are frequently transient. VEGFR-2 inhibition is noteworthy because it increases hypoxic stress, which stabilizes HIF-1α and triggers compensatory mechanisms, including the c-Met signaling pathway [6].

Hepatocyte growth factor (HGF) receptor tyrosine kinase c-Met is another important modulator of tumor angiogenesis, invasion, and metastasis. Phosphorylation events triggered by c-Met activation initiate downstream signaling pathways associated with tumor growth and metastasis [7]. Since resistance to VEGFR-targeted treatments has been associated with overexpression of c-Met, it is an essential supplementary target [8]. Numerous selective c-Met inhibitors have been developed (Figure 1), such as savolitinib (in clinical trials for renal cell carcinoma), capmatinib and tepotinib (authorized for MET exon 14-altered NSCLC), and crizotinib (initially an ALK/c-Met inhibitor, FDA-approved for NSCLC) [9].

Dual inhibition of both kinases has become a viable anticancer treatment [10] due to the substantial interaction between VEGFR-2 and c-Met, which increases angiogenesis and contributes to therapeutic resistance (Figure 1). With FDA approval for both renal cell carcinoma and medullary thyroid cancer, cabozantinib is one of the most therapeutically important dual inhibitors. It exhibits strong action against both targets [11]. Other noteworthy examples are foretinib (XL880) and golvatinib (E7050), which are being studied in clinical settings and have shown dual VEGFR-2/c-Met inhibition [8]. These drugs demonstrate how dual targeting can be used therapeutically to overcome medication resistance and enhance solid tumor outcomes.

Our derivatives’ logical design was founded on the idea of creating dual VEGFR-2 and c-Met inhibitors by combining the crucial pharmacophoric characteristics needed for both kinases. It is anticipated that combined inhibition of these two targets may result in greater therapeutic success than single-target medicines because they are crucial for tumor development, angiogenesis, and metastasis. Three key pharmacophoric components are shared by sorafenib and other clinically significant inhibitors of VEGFR-2 [12]. The adenosine triphosphate (ATP)-binding domain accommodates the first, flat heteroaromatic ring. The second is a core hydrophobic ring that is joined to a hydrogen-bonding moiety (such as an amide or urea group) and functions as both a hydrogen bond acceptor (HBA) and donor (HBD), allowing interactions with two important residues in the kinase domain: Glu885 and Asp1046. In particular, the C=O group interacts with Asp1046, whereas the NH motif typically forms a hydrogen bond with Glu885. Strong ligand stability within the active site is ensured by the third member, a terminal hydrophobic moiety that extends into and occupies the allosteric hydrophobic pocket.

Cabozantinib is a reference inhibitor that demonstrates the structural prerequisites for efficient inhibition, and c-Met has also been confirmed as a viable therapeutic target [13]. A phenoxy linker attached to a cyclopropane-1,1-dicarboxamide fragment that sustains a strong hydrogen bond with the hinge region, a 4-fluorophenyl moiety that provides hydrophobic stabilization within a lipophilic sub-pocket, and a quinoline moiety that functions as a planar aromatic head to occupy the ATP-binding site are some of its pharmacophoric characteristics. Together, these characteristics enable improved selectivity, hinge stabilization, and effective occupation of the ATP-binding site.

**Figure 2:**
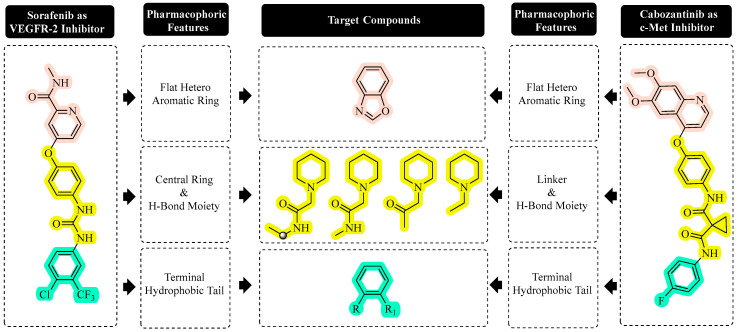
Rationale of design for dual c-Met/VEGFR-2 inhibitors.

Our compounds were designed to combine the structural and pharmacophoric features necessary for dual inhibition of VEGFR-2 and c-Met. The benzoxazole scaffold was selected as a planar heteroaromatic core to facilitate ATP-pocket binding and π–π interactions, while the central piperidine ring serves as a flexible hydrophobic linker that optimizes positioning of functional groups for hydrogen bonding and lipophilic interactions. This hybrid design was intended to enhance binding affinity, selectivity, and overall anticancer activity, providing a rationale for choosing the benzoxazole-piperidine scaffold in the current study (Figure 2).

All things considered, this logical design approach guarantees that the novel derivatives satisfy the pharmacophoric requirements of both c-Met inhibition and VEGFR-2 inhibition. In comparison to current single-target inhibitors, the newly created compounds are anticipated to exhibit strong dual-target activity by fusing the structural characteristics of sorafenib and cabozantinib within a single molecular framework. This could lead to improved pharmacokinetic characteristics and increased therapeutic efficacy.

## 2. Results and Discussion

### 2.1. Chemistry

**Scheme 1:**
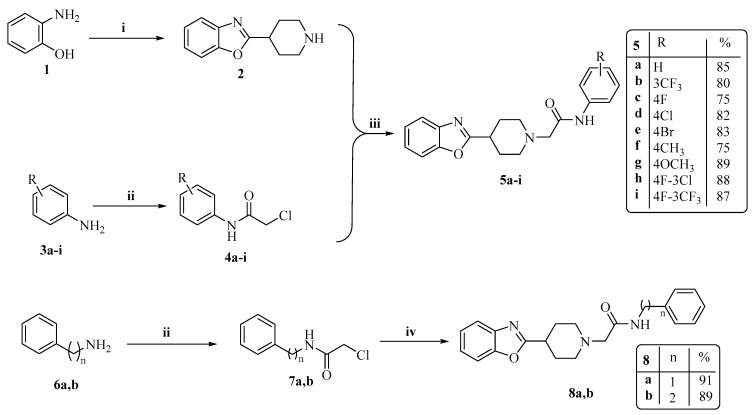
Synthesis of the key intermediates **2**, **4a**–**i** and **7a**,**b** and target compounds **5a**–**i** and **8a**,**b**: Reagents and conditions (i) Piperidine-4-carboxylic acid, PPA at 180 °C for 2 h; (ii) Chloroacetyl chloride, TEA, dioxane, stirring ice bath, 2 h; (iii) Acetone, K_2_CO_3_, KI, reflux 4 h; (iv) Compound **2**, acetone, K_2_CO_3_, KI, reflux 4 h.

**Scheme 2:**
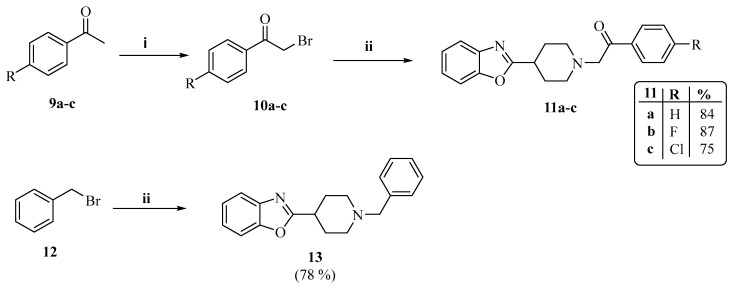
Synthesis of the key intermediates **10a**–**c**, and target compounds **11a**–**c** and **13**: Reagents and conditions (i) Chloroform (20 mL), acetonitrile (5 mL), NBS, reflux, 4 h; (ii) Compound **2**, acetone, K_2_CO_3_, KI, reflux 4 h.

The synthetic pathway for novel piperidinyl-based benzoxazole derivatives **5a**–**i**, **8a**,**b**, **11a**–**c**, and **13** was accomplished, as depicted in Scheme 1 and Scheme 2. 2-Aminophenol **1** was refluxed with piperidine-4-carboxylic acid in the presence of polyphosphoric acid (PPA), undergoing cyclization to yield the desired intermediate, 2-(piperidin-4-yl)benzo[*d*]oxazole **2** [14]. Different aromatic amines **3a**–**i** and aliphatic amines **6a**,**b** were treated with chloroacetyl chloride to prepare the corresponding intermediates **4a**–**i** and **7a**,**b** in the presence of TEA [15,16]. The final target acetamides **5a**–**i** (75–89% yield) and **8a**,**b** (89–91% yield) were prepared in acetone via nucleophilic substitution through the reaction of **4a**–**i** and **7a**,**b** with 2-(piperidin-4-yl)benzo[*d*]oxazole **2,** utilizing K_2_CO_3_ and KI as a base catalyst, Scheme 1.

Additionally, the freshly prepared phenacyl bromides **10a**–**c** (obtained from acetophenone derivatives **9a**–**c** via NBS in a hot mixture of chloroform and acetonitrile) were stirred with intermediate 2 in acetone, utilizing K_2_CO_3_ and KI as a base catalyst. After completion, the reaction mixture was poured into crushed ice to yield the corresponding final candidates **11a**–**c** (75–87% yield). Moreover, 2-(piperidin-4-yl)benzo[*d*]oxazole **2** was reacted with benzyl bromide **12** in acetone utilizing K_2_CO_3_ and KI as a base catalyst for 4 h to afford the 2-(1-benzylpiperidin-4-yl)benzo[*d*]oxazole **13** (78% yield), Scheme 2.

Spectral analyses confirmed the successful synthesis of the piperidinyl benzo[*d*]oxazole derivatives. In the case of the acetamide derivatives (**5a**–**i** and **8a**,**b**), the NMR spectra (Figures S1–S28) displayed the anticipated characteristic signals, including the protons and carbons of the piperidine ring in the aliphatic region, the CH_2_CO fragment appearing at 2.84–3.15 ppm in ^1^H NMR and 62.02–62.55 ppm in ^13^C NMR, and the NH resonance at 9.55–10.10 ppm for compounds **5a**–**i** and 7.71–8.31 ppm for compounds **8a**,**b**. Likewise, for the target derivatives (**11a**–**c** and **13**), a distinct signal corresponding to the CH_2_ group was observed at 3.45–3.83 ppm, accompanied by characteristic carbonyl resonances in the ^13^C NMR spectra, together with additional peaks attributed to aliphatic carbons.

Within the permitted range (±0.4), the molecular formula of the target compounds closely matched the elemental analysis results. Collectively, these spectroscopic data strongly support the proposed structures of the synthesized compounds, as further described in Section 3.

### 2.2. Biological Activity

#### 2.2.1. Enzyme Assay

Three series were used to classify all newly created piperidinyl-based benzoxazole derivatives: ethanone derivatives (**11a**–**c**), alkyl derivatives (**13**), and acetamide derivatives (**5a**–**i** and **8a**,**b**). Table 1 summarizes the findings of an evaluation of these compounds’ inhibitory action against the c-Met and VEGFR-2 kinases. The measured IC_50_ values for c-Met and VEGFR-2 varied from 0.181 to 2.210 µM and 0.057 to 1.680 µM, respectively.

Positive controls included the well-known kinase inhibitors staurosporine (IC_50_ = 0.237 µM, c-Met) and sorafenib (IC_50_ = 0.058 µM, VEGFR-2). Interestingly, the most active substances were found to be the ethanone derivatives **11a** and **11b**. Compound **11b**, which contains a *p*-fluorophenyl moiety, demonstrated superior activity with IC_50_ values of 0.057 µM (VEGFR-2) and 0.181 µM (c-Met), putting it on par with sorafenib against VEGFR-2 and even surpassing staurosporine against c-Met. Compound **11a**, which contains a plain phenyl moiety, inhibited VEGFR-2 and c-Met with IC_50_ values of 0.082 and 0.280 µM, respectively.

With IC_50_ values of 2.210 µM (VEGFR-2) and 1.514 µM (c-Met), the acetamide derivative **5i**, which contains a *p*-fluoro-*m*-trifluoromethylphenyl moiety, was found to be the least effective. With the exception of compounds **5b**, **5e**, and **8a**, which demonstrated comparatively greater efficacy against c-Met, a comparison of the two kinase targets showed that the majority of compounds showed better inhibitory activity against VEGFR-2 than against c-Met.

**Figure 3:**
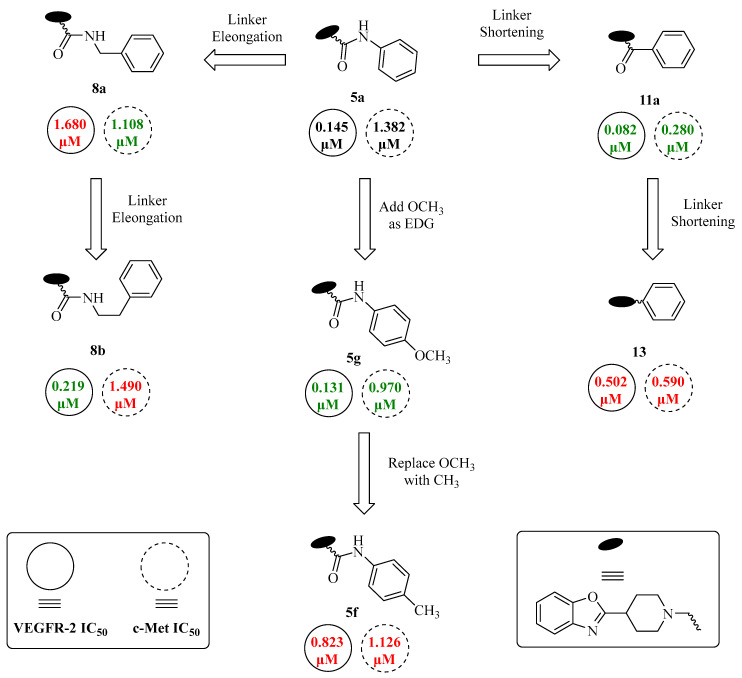
Potential inhibitory effect of unsubstituted phenyl derivatives and substituted derivatives with EDG on dual VEGFR-2 and c-Met. Red color indicates decreased activity, while green color indicates increased activity.

With IC_50_ values of 0.145 µM (VEGFR-2) and 1.382 µM (c-Met), the unsubstituted phenyl derivative **5a** (acetamide series) demonstrated modest dual inhibitory activity. The inhibitory action against c-Met was somewhat enhanced (1.108 µM) but significantly decreased against VEGFR-2 (1.680 µM) with linker elongation by substituting a benzyl moiety (**8a**) for the phenyl ring. In comparison to **8a**, further elongation by adding an ethylphenyl linker (**8b**) resulted in a considerable increase in activity against VEGFR-2 (0.219 µM) but a decrease in potency against c-Met (1.490 µM). The inhibitory action against both targets was significantly increased by linker shortening by changing the acetamide linker into an ethanone linker (**11a**), which produced IC_50_ values of 0.082 µM (VEGFR-2) and 0.280 µM (c-Met) in comparison to **5a**. However, compound **13**, which further shortens the linker by eliminating the carbonyl group, lost its activity against both kinases (0.502 µM, VEGFR-2; 0.590 µM, c-Met). These results suggest that excessive shortening reduces activity; however, the ethanone linker is ideal for achieving dual inhibitory efficacy (Figure 3).

Activity was greatly affected by substituting electron-donating groups (EDGs) in the *para* position for the phenyl ring in the acetamide series. When compared to the unsubstituted counterpart **5a**, compound **5g**’s addition of a *para*-methoxy group increased activity against both kinases (0.131 µM, VEGFR-2; 0.970 µM, c-Met). However, compound **5f**, which substitutes a less polar methyl substituent for the methoxy group, decreases the inhibitory activity against both VEGFR-2 (0.823 µM) and c-Met (1.126 µM) compared to compound **5g**, demonstrating the methoxy substituent’s higher electronic and steric contributions (Figure 3).

**Figure 4:**
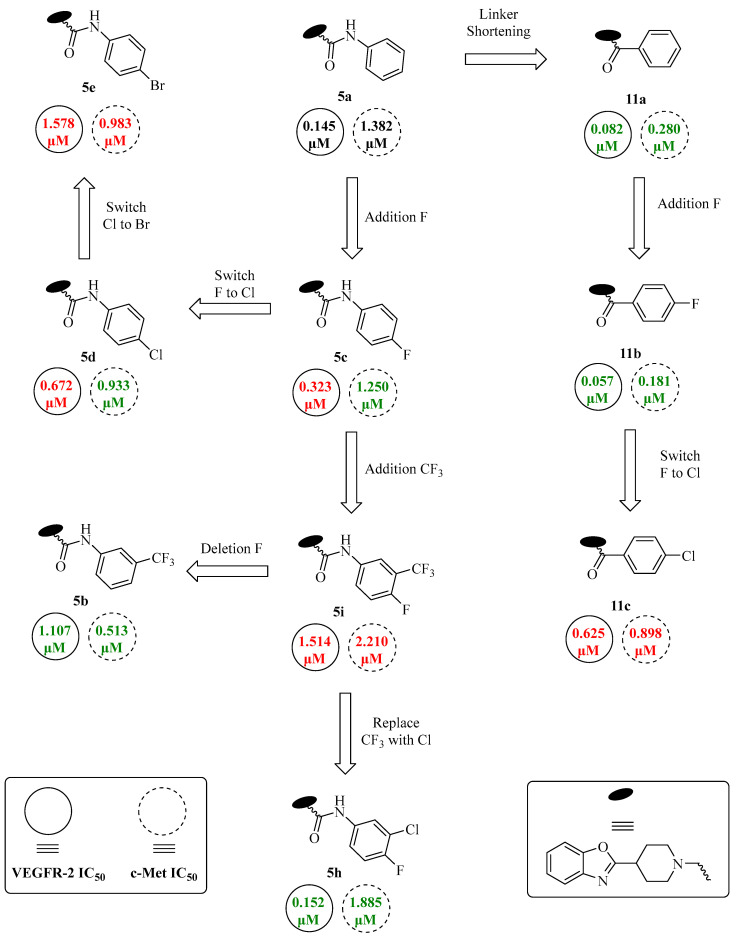
Potential inhibitory effect of halogenated substitutions on dual VEGFR-2 and c-Met. Red color indicates decreased activity, while green color indicates increased activity.

The addition of a fluorine atom at the *para* position (**11b**) considerably increased the inhibitory activity against both kinases in halogenated compounds, beginning with the unsubstituted analogue **11a** (plain phenyl moiety), yielding IC_50_ values of 0.057 µM (VEGFR-2) and 0.181 µM (c-Met) in comparison to **11a** (0.280 and 0.082 µM, respectively). However, a significant decrease in activity against both targets (0.898 µM, c-Met; 0.625 µM, VEGFR-2) was observed when a bulkier chlorine atom was substituted for fluorine (**11c**), indicating that steric hindrance at the *para* position had a detrimental effect on binding affinity (Figure 4).

The parent compound **5a** (plain phenyl moiety) exhibited modest inhibitory activity (0.145 µM, VEGFR-2; 1.382 µM, c-Met) for the acetamide derivatives. A *para*-fluorine atom (**5c**) was substituted, which decreased VEGFR-2 efficacy (0.323 µM vs. 0.145 µM) but increased c-Met inhibition (1.250 µM vs. 1.382 µM). In comparison to **5c**, substituting the bigger chlorine atom (**5d**) for fluorine increased c-Met activity (0.933 µM) but decreased VEGFR-2 inhibition (0.672 µM). Reduced activity against both kinases (0.983 µM, c-Met; 1.578 µM, VEGFR-2) was obtained by substituting an even bulkier bromine atom (**5e**), confirming the negative steric effect of bigger halogens (Figure 4).

Going back to compound **5c** (*para*-fluoro derivative), the addition of a *meta*-CF_3_ substituent (**5i**) significantly reduced activity against both c-Met (2.210 µM) and VEGFR-2 (1.514 µM) in comparison to compound **5c**. This suggests that the large, electron-withdrawing CF_3_ group at the *meta* position is not advantageous. Interestingly, activity was restored when the *para*-fluorine from **5i** was removed while the *meta*-CF_3_ (compound **5b**) was left in place. This improved the inhibition of both VEGFR-2 (1.107 µM) and c-Met (0.513 µM) in comparison to **5i**. Additionally, adding a chlorine atom (**5h**) to **5i**’s CF_3_ group increased activity, especially against VEGFR-2 (0.152 µM), and slightly increased c-Met inhibition (1.885 µM) compared to **5i**.

#### 2.2.2. In Vitro Antiproliferative Activity and Selectivity

The cytotoxic efficacy of the most potent compounds (**5a**, **5g**, **5h**, **11a**, and **11b**) against three human tumor cell lines, such as MCF-7 (breast cancer), A549 (lung cancer), and PC-3 (prostate cancer), was assessed using the MTT test. Based on IC_50_ values, their activities were contrasted with those of the reference medication, sorafenib (Table 2). With better results against the breast cancer cell line (MCF-7) than the lung (A549) and prostate (PC-3) cancer cells, all tested derivatives reduced the proliferation of the three cancer cell lines in a dose-dependent manner.

When tested against the tested cell lines, compounds from the ethanone series (**11a** and **11b**) showed single-digit micromolar IC_50_ values (with the exception of **11a** against PC-3). With IC_50_ values of 4.30, 6.68, and 7.06 µM, respectively, compound **11b** (containing a *p*-fluorophenyl moiety) demonstrated strong cytotoxic action against MCF-7, A549, and PC-3 cells. These values are very similar to those of sorafenib (4.95, 6.32, and 6.57 µM, respectively). IC_50_ values of 6.25, 8.33, and 15.95 µM, respectively, demonstrated good cytotoxic action as well as compound **11a**, which carries a plain phenyl moiety. With an effectiveness comparable to sorafenib, these findings demonstrate compound **11b** as a strong and selective anticancer candidate against breast cancer (MCF-7).

However, with the exception of compound **5g** against A549, which reached the single-digit micromolar range, compounds of the acetamide series (**5a**, **5g**, and **5h**) typically displayed two-digit micromolar IC_50_ values. The IC_50_ values of compound **5a** (plain phenyl moiety) against MCF-7, A549, and PC-3 were 16.29, 23.60, and 16.14 µM, respectively. Compound **5h** (*p*-fluoro *m*-chlorophenyl moiety) obtained IC_50_ values of 14.01, 14.86, and 24.89 µM against the same cell lines, while compound **5g** (*p*-methoxyphenyl moiety) showed IC_50_ values of 17.23, 9.32, and 22.63 µM.

All of these results highlight the encouraging cytotoxic potential of ethanone derivatives, especially that of compound **11b**, which is a prime contender for further advancement in breast cancer treatment.

#### 2.2.3. Cytotoxic Effects In Vitro on MCF-10A (Normal Cells)

One well-known disadvantage of many anticancer medications is their inability to distinguish between malignant and non-malignant cells, which frequently results in dose-limiting toxicities [17]. Consequently, the most active derivatives’ cytotoxic effects (**5a**, **5g**, **5h**, **11a**, and **11b**) were evaluated further against the normal breast epithelial cell line MCF-10A to ascertain their selectivity indices (SI), which are determined by dividing their IC_50_ on MCF-10A by that on MCF-7 cells (Table 2).

With SI values near 2, compounds from the acetamide series (**5a**, **5g**, and **5h**) demonstrated comparatively poor selectivity. For example, compound **5g** (*p*-methoxyphenyl moiety) and compound **5h** (*p*-fluoro-*m*-chlorophenyl moiety) showed SI values of 1.76 (30.35/17.23 µM) and 1.99 (27.85/14.01 µM), respectively, whereas compound **5a** (plain phenyl moiety) showed an SI of 1.74 (28.31/16.29 µM). These findings suggest that the acetamide derivatives have similar cytotoxic effects on breast cancer cells and healthy cells, indicating a lack of discernible selectivity.

On the other hand, substances from the ethanone class showed noticeably better selectivity for MCF-7 cells. Compound **11b** (*p*-fluorophenyl moiety) obtained the greatest SI value of 7.97 (34.25/4.30 µM), while compound **11a** (plain phenyl moiety) displayed an SI of 4.35 (27.17/6.25 µM). Interestingly, **11b**’s SI was almost twice as selective for breast cancer cells than non-malignant breast epithelial cells, outperforming the reference medication sorafenib (SI = 4.37, 21.63/4.95 µM).

When considered collectively, these results illustrate the superiority of ethanone derivatives, especially compound **11b**, which not only showed strong cytotoxicity but also a significant level of selectivity towards malignant breast cells when compared to the sorafenib and acetamide analogues. This improved selectivity highlights **11b**’s potential as a viable lead contender for additional advancements in breast cancer treatment.

#### 2.2.4. Cell Cycle Analysis in MCF-7 Cells Treated with **11b**

Cell cycle analysis is a crucial technique in cancer research because it provides insight into how potential anticancer medications inhibit the proliferation of tumor cells [18]. Cell division and development are governed by the G_1_, S, G_2_, and M stages of the cell cycle [19]. One of the hallmarks of cancer is cell cycle dysregulation, leading to uncontrolled cell growth. Therefore, to stop tumor development and induce cell-cycle arrest, several anticancer medications target specific checkpoints [20].

**Figure 5:**
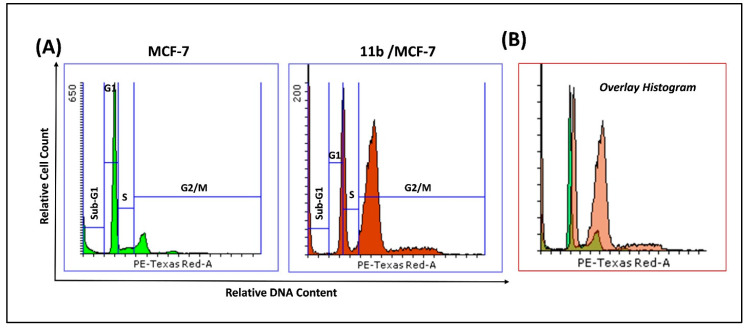
Flow cytometric analysis of cell cycle phases post-treatment of the cells with the tested compound **11b** for 72 h. (**A**) Cell cycle analysis was performed by flow cytometry. (**B**) Overlay histogram of the representative histograms compound compared to the control after 72 h treatment.

Compound **11b**’s impact on MCF-7 cells’ cell-cycle distribution was assessed (Figure 5). As demonstrated by a significant decrease in the percentage of G_1_-phase cells (from 57.59% to 10.43%, Δ −47.16) and an increase in cells in the S-phase (from 7.37% to 13.90%, Δ +6.53) and the G_2_/M phase (from 23.22% to 57.82%, Δ +34.60), treatment with **11b** significantly changed the cell-cycle profile when compared to the untreated control. These results demonstrate that **11b** effectively inhibits DNA synthesis and induces a robust G_2_/M phase arrest, thereby disrupting the normal cell-cycle progression of MCF-7 cells.

#### 2.2.5. Annexin V-FITC/Propidium Iodide (PI) Assay for Compound **11b** in MCF-7 Cells

**Figure 6:**
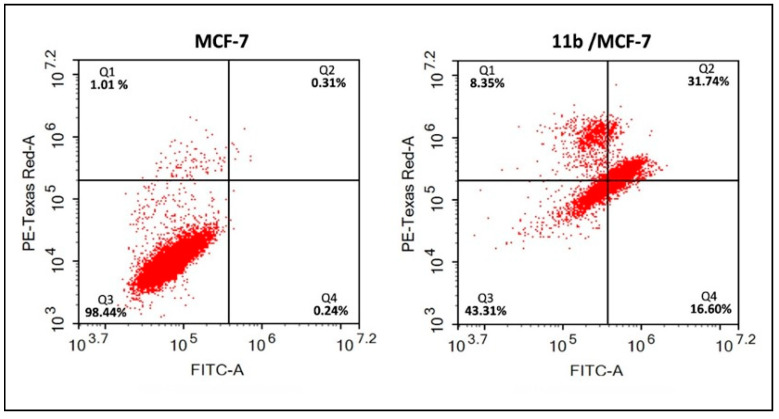
Flow cytometric analysis of apoptosis in the tested compound **11b** on MCF-7 cells treated for 72 h. Representative scatter plots of PI vs. annexin V showing the images and percentages of cell apoptosis in MCF-7 (**left**) and compound **11b**-treated cells (**right**).

Apoptosis, also known as programmed cell death, is a vital process that maintains cellular homeostasis and prevents uncontrolled cell division [21]. Cancer often disrupts apoptotic signaling pathways, which enables tumor cells to avoid cell death and continue to grow [22]. Therefore, one of the most important treatment strategies in the hunt for novel anticancer medications is to induce tumor cells to undergo apoptosis again [23]. The Annexin V-FITC/Propidium Iodide (PI) assay is commonly used to evaluate apoptosis because it can differentiate between necrotic cells (Annexin V-negative/PI-positive), viable cells (Annexin V-negative/PI-negative), late apoptotic cells (Annexin V-positive/PI-positive), and early apoptotic cells (Annexin V-positive/PI-negative) [24]. This enables the accurate assessment of how compound **11b** compounds induce cell death (Figure 6).

Comparing compound **11b**-treated MCF-7 cells to the untreated control, a notable change in cell fate was observed. Exposure to **11b** significantly altered the cell distribution in the control group, which was primarily composed of viable cells (98.44%) with very low levels of necrosis (1.01%), late apoptosis (0.31%), and early apoptosis (0.24%). In particular, there was a significant drop in cell viability to 43.31% as early apoptosis rose to 16.60%, late apoptosis to 31.74%, and necrosis to 8.35%. These data unequivocally demonstrate that compound **11b** primarily induces apoptosis, rather than necrosis, with early apoptosis emerging as the primary mechanism of cell death. The conclusion that apoptosis induction is a key mechanism behind **11b**’s anticancer activity in MCF-7 cells is highly supported by the significant percentage of apoptotic cells.

#### 2.2.6. Effects of Compound **11b** on the Apoptotic Markers

In MCF-7 breast cancer cells, the impact of compound **11b** on the transcriptional regulation of the primary apoptotic markers (p53, caspase-9, Bcl-2, and BAX) was examined. qRT-PCR was used to measure the levels of gene expression after 72 h of treatment with **11b** at a dose of 4.30 μM (Table S1). The findings showed that both BAX and caspase-9 were significantly upregulated, with each exhibiting a 2.48-fold increase compared to the untreated control. The anti-apoptotic gene Bcl-2, on the other hand, was significantly downregulated, exhibiting a twofold reduction compared to control cells. Interestingly, after **11b** treatment, p53 expression increased significantly, rising from a normalized baseline of 1.00 in untreated cells to 4.56 times the baseline.

**Figure 7:**
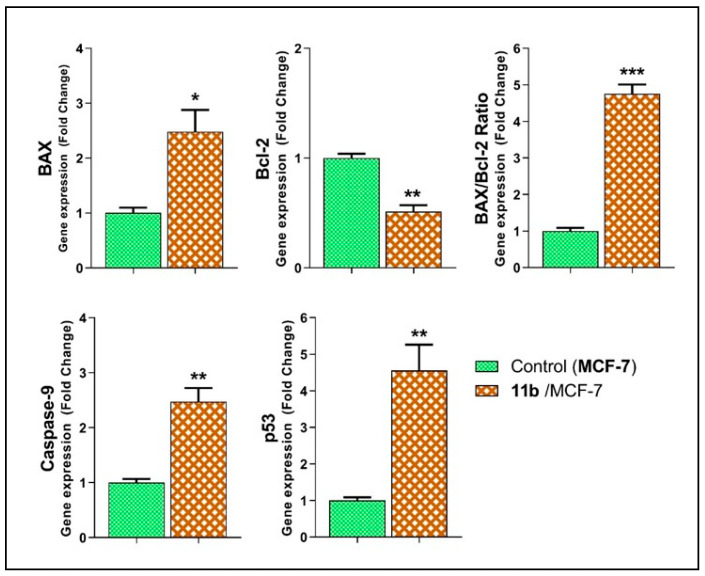
Gene expression study for BAX, Bcl-2, cleaved caspase-9, and p53 expression levels following 72 h of treatment with MCF-7 at an IC_50_ dose of **11b**. After being planted in tissue culture flasks, MCF-7 cells were exposed to **11b** for 72 h. To evaluate the expression of the BAX, Bcl-2, caspase-9, and p53 genes, total RNA was collected and reverse-transcribed using quantitative reverse transcription-PCR. The housekeeping gene is GAPDH. The mean ± SEM was used to indicate the ratio of target gene/GAPDH expression (relative mRNA levels) derived from at least three separate studies. Fold changes were used to express normalized data, and “1” was used as the control. In unpaired *t*-tests, statistically significant differences from the matching control are indicated by * *p* < 0.05, ** *p* < 0.01, and *** *p* < 0.001.

The significant increase in the BAX/Bcl-2 ratio, which went from 1.00 in control cells to 4.76 after treatment, was a particularly significant observation. Given that this ratio is a crucial factor in mitochondrial-mediated apoptosis, its dramatic rise demonstrates **11b**’s strong pro-apoptotic effects. When considered as a whole, these molecular alterations provide compelling evidence that compound **11b** stimulates apoptosis by activating the intrinsic (mitochondrial) apoptotic pathway, thereby confirming its potential as an anticancer option against MCF-7 breast cancer cells (Figure 7, Table 3 and Table S1).

To evaluate the kinase selectivity of the target compounds, the compound **11a** was screened at 10 µM against a panel of 6 kinases, and the percentage inhibition for each enzyme was measured (Table 4). The results revealed that compound **11a** did not exhibit notable inhibition toward any of the tested kinases. Overall, these findings indicate that **11a** possesses favorable selectivity for VEGFR-2 kinase.

### 2.3. Molecular Modeling Study

In the current work, we performed molecular docking studies to better understand how our new compounds interacted with the target proteins. Specifically, we docked the synthesized derivatives into the ATP-binding sites of VEGFR-2 (PDB: 4ASD) and c-Met (PDB: 4R1V) to determine whether their binding modes could explain the observed enzymatic inhibition. Our analysis focused on the two most potent compounds, **11a** and **11b**, exploring how they fit within the active sites and identifying the key molecular interactions that are likely to contribute to their potent inhibitory activities.

#### 2.3.1. Molecular Docking of Compounds **11a** and **11b** Against VEGFR-2 TK

**Figure 8:**
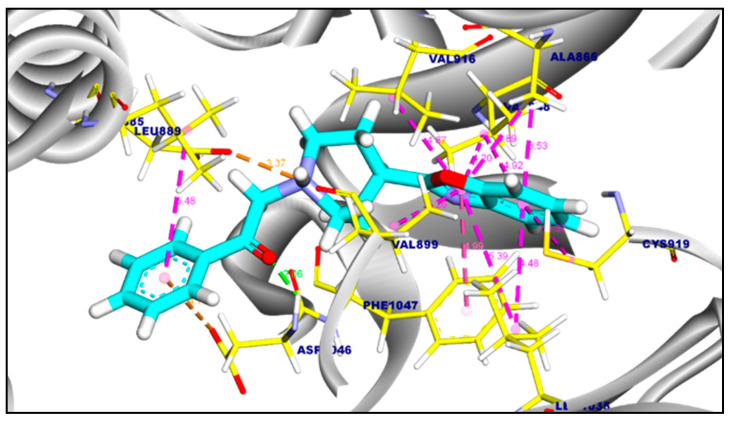
3D illustration of compound **11a**’s suggested binding mechanism against VEGFR-2 TK. The side chain of an amino acid is colored yellow, whereas the tested molecule is colored turquoise.

**Figure 9:**
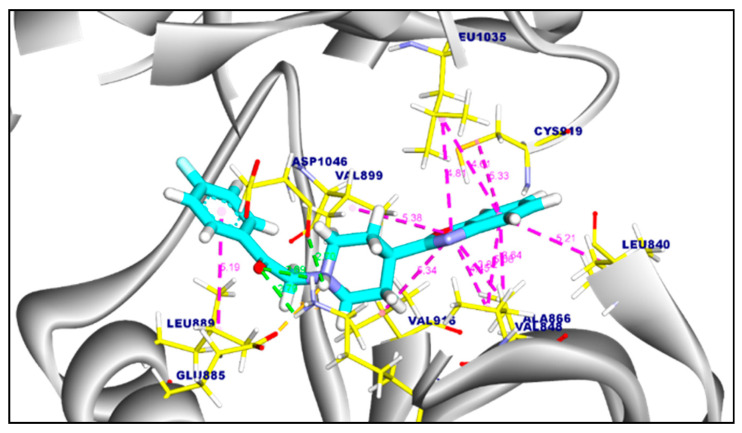
3D illustration of compound **11b**’s suggested binding mechanism targeting VEGFR-2 TK. The side chain of an amino acid is colored yellow, whereas the tested molecule is colored turquoise.

The proposed binding mode of compound **11a** exhibited an affinity binding energy of −8.57 kcal/mol against VEGFR-2 TK. The benzo[*d*]oxazol-2-yl moiety interacted through nine hydrophobic π–alkyl and one π–π interactions with Leu1035, Cys919, Ala866, Val916, Val848, Val899, and Phe1047. Additionally, hydrogen bonds and ionic interactions were observed with the pharmacophoric amino acids Asp1046 (2.06 Å) and Glu885. Additionally, the terminal phenyl moiety formed two π–alkyl and π–anion interactions with the side chains of Leu889 and Asp1046 (Figure 8 and Table 5). Moreover, with the same pattern of interaction, compound **11b** exhibited an affinity score of −8.62 kcal/mol against VEGFR-2 TK. The benzo[*d*]oxazol-2-yl moiety and terminal *p*-fluorophenyl fragment formed eleven hydrophobic π–alkyl interactions with Leu840, Cys919, Leu1035, Val848, Val916, Ala866, Val899, and Leu889. Furthermore, the interaction was supported by two strong hydrogen bonds and ionic interaction with Asp1046 (2.70 Å), Lys868 (2.74 Å), and Glu885 (Figure 9 and Table 5).

**Figure 10:**
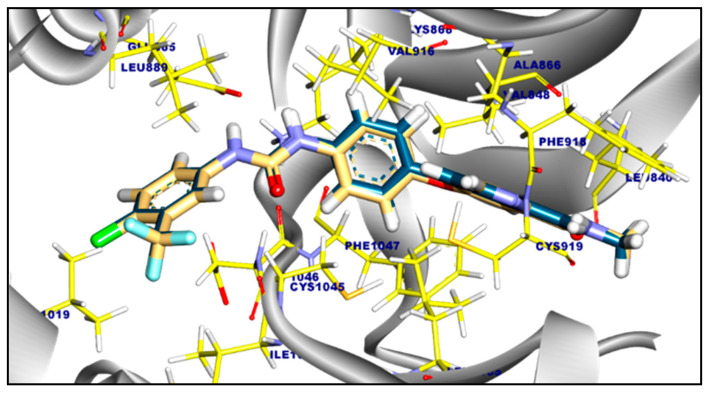
Sorafenib, the co-crystallized ligand complexed with VEGFR-2 TK, is superimposed. The redocked pose of sorafenib, which has an RMSD value of 0.15 Å, is colored dark blue, while the original stance is colored faint brown.

**Figure 11:**
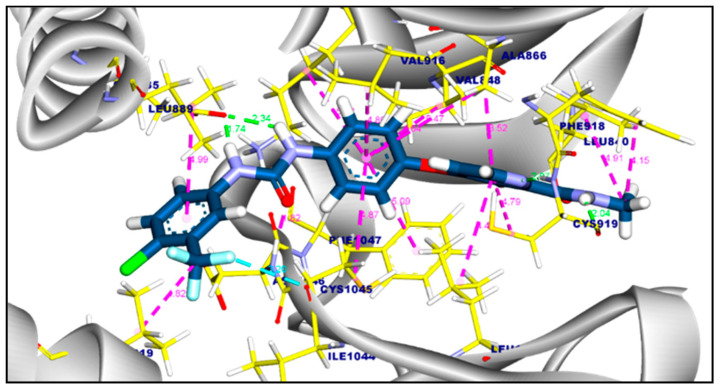
An illustration of the co-crystallized ligand sorafenib complexed with VEGFR-2 TK and its suggested binding mechanism. The side chain of an amino acid is colored yellow, while sorafenib is dark blue.

On the other hand, the co-crystalized ligand complexed with VEGFR-2 TK (Sorafenib) exhibited an affinity score of −9.18 kcal/mol with an RMSD value of 0.15 Å, which indicates that the docking process was valid (Figure 10 and Table 5). Sorafenib formed 13 hydrophobic π–alkyl and π–π interactions with Phe918, Leu840, Phe1047, Val916, Val848, Lys868, Leu889, Ile1044, Leu1019, Cys1045, Ala866, and Cys919, in addition to five hydrogen bonds with essential pharmacophoric amino acids side chain as Cys919, Asp1046, and Glu885 with distances of 2.04, 2.07, 1.82, 2.34 and 1.74 Å, respectively (Figure 11 and Table 5).

#### 2.3.2. Molecular Docking of Compound **11a** and **11b** Against c-Met Kinase

**Figure 12:**
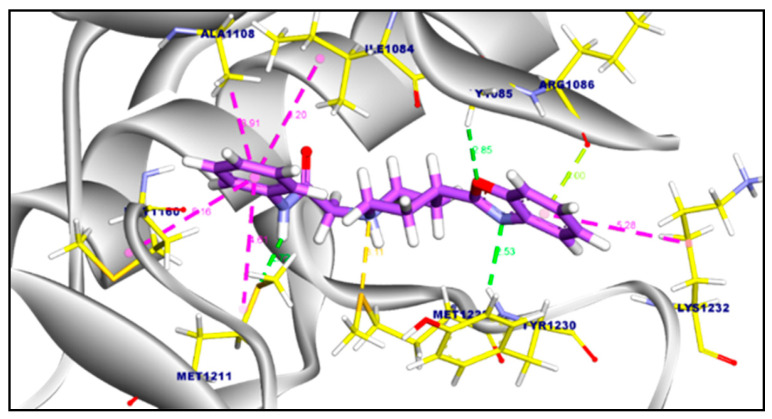
3D illustration of chemical **11a**’s suggested binding mechanism to c-Met kinase. The side chain of an amino acid is colored yellow, whereas the tested compounds are tinted violet.

The binding mode of compound **11a** displayed an affinity score of −7.98 kcal/mol against c-Met kinase. Two hydrophobic interactions, π–alkyl and π–lone pair, were observed between the benzo[*d*]oxazol-2-yl moiety and Lys1232 and Arg1086. Additionally, the terminal phenyl ring formed five hydrophobic π–alkyl and π–sulfur interactions with Ala1108, Met1211, Ile1084, and Met1160. Moreover, compound **11a** established three hydrogen bonds with Tyr1230, Met1211, and Gly1085, at distances of 2.53, 2.72, and 2.85 Å, respectively (Figure 12 and Table 5).

**Figure 13:**
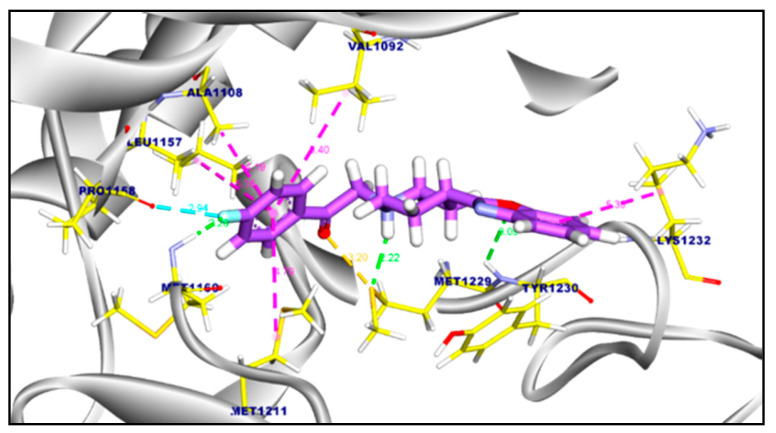
3D illustration of compound **11b**’s suggested binding mechanism to c-Met kinase. The side chain of an amino acid is colored yellow, whereas the tested molecule is tinted violet.

In comparison, the proposed binding mode of compound **11b** exhibited an affinity score of −7.93 kcal/mol against c-Met kinase. Compound **11b** formed multiple hydrophobic π–alkyl and π–sulfur interactions with Lys1232, Met1229, Met1211, Ala1108, Val1092, and Leu1157. Additionally, it showed two hydrogen bonds to Tyr1230 and Met1229, with bond distances of 3.09 and 2.22 Å, respectively. Furthermore, the *p*-fluoro substituent in the terminal hydrophobic moiety formed an additional hydrogen bond and a halogen interaction with residues Met1160 and Pro1158, exhibiting bond distances of approximately 2.28 Å. These interactions likely contribute to stronger, more stable binding of compound **11b** within the target pocket compared with the other tested candidates (Figure 13 and Table 5).

**Figure 14:**
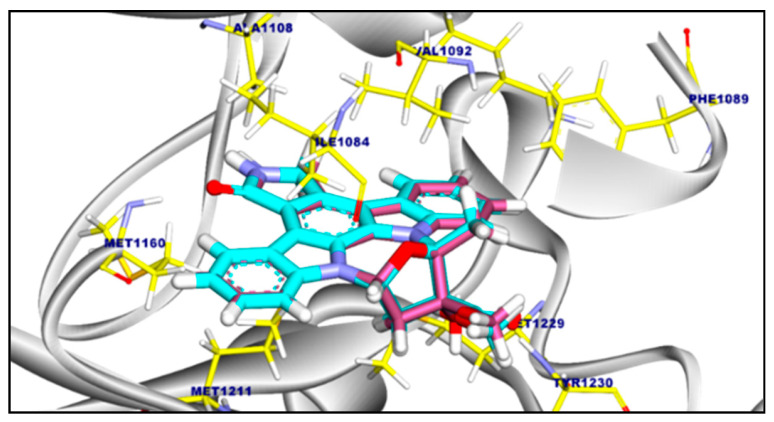
Superimposition of the c-Met kinase complexed with the co-crystallized ligand (staurosporine). With an RMSD of 0.18 Å, the staurosporine’s original stance was colored turquoise, and its redocked pose was colored purple.

**Figure 15:**
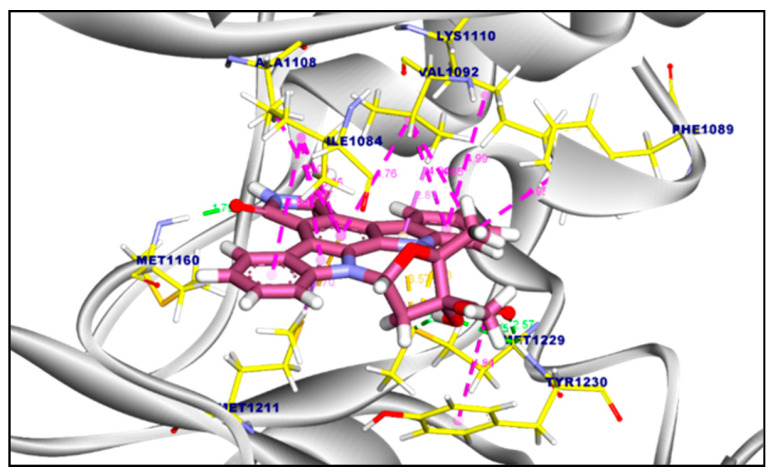
An illustration of the co-crystallized ligand (staurosporine) complexed with c-Met kinase and its suggested binding mechanism. The side chain of an amino acid is colored yellow, whereas the staurosporine is purple.

Meanwhile, the co-crystallized ligand staurosporine, complexed with c-Met kinase, demonstrated an affinity score of −9.27 kcal/mol with an RMSD value of 0.18 Å, confirming the reliability of the docking protocol (Figure 14 and Table 5). Staurosporine was stabilized by fifteen hydrophobic interactions, including π–alkyl, π–sulfur, and π–sigma contacts with Ile1084, Met1211, Tyr1230, Phe1089, Met1229, Lys1110, Val1092, and Ala1108. In addition, three hydrogen bonds were observed with Met1160, Tyr1230, and Met1229, at distances of 1.79 Å, 2.57 Å, and 2.23 Å, respectively (Figure 15 and Table 5).

It is worth mentioning that the flexibility afforded by rotation around the piperidine N–C bond allowed the inhibitors to navigate the steric constraints of the active site and adopt a conformation compatible with the DFG-out motif. This conformational adaptability permitted the terminal hydrophobic fragment to penetrate deeply beyond the gatekeeper residues (Leu889 in VEGFR-2 and Met1160 in c-Met), thereby stabilizing the inhibitor within the allosteric hydrophobic pocket.

The docking protocol was validated by redocking co-crystallized ligands, yielding RMSD values of 0.15 Å (sorafenib/VEGFR-2) and 0.18 Å (staurosporine/c-Met), well below the 2.0 Å threshold. Compounds **11a** and **11b** exhibited RMSD values of 0.94–1.36 Å relative to the references (Table 5); while RMSD comparisons between distinct structures have inherent limitations, these results confirm that our compounds adopt binding orientations analogous to clinical inhibitors. Regarding scores, marginal differences (e.g., −8.57 vs. −8.62 kcal/mol) fell within standard error margins and are interpreted cautiously, prioritizing qualitative validation over numerical ranking. Notably, the conserved interactions with key residues (Asp1046/Glu885 for VEGFR-2; Tyr1230/Met1160 for c-Met) structurally rationalize the potent enzymatic inhibition observed for 11b (IC_50_: 0.057 μM VEGFR-2, 0.181 μM c-Met).

## 3. Materials and Methods

### 3.1. Chemistry

#### 3.1.1. General

Chemical shifts (***δ***) were reported in parts per million (ppm) with respect to the solvent peak in NMR spectra obtained on a JEOL Resonance spectrometer (JEOL Ltd., Tokyo, Japan) that operated at 500 MHz for ^1^H and 126 MHz for ^13^C nuclei. The solvent used was deuterated dimethyl sulfoxide (DMSO-*d*_6_). The unit of measurement for coupling constants (*J*) is Hertz (Hz). Melting points are presented uncorrected and were measured using a Stuart melting point instrument. Unless otherwise noted, all reagents and solvents were purchased from commercial vendors and utilized without additional purification. According to documented protocols, the intermediates **2** [14], **4a**–**i** [25], **7a**,**b** [26,27], and **10a**–**c** [28] were previously synthesized.

#### 3.1.2. The General Synthesis of Target Piperidinyl-Based Benzoxazole Derivatives (**5a**–**i**, **8a**,**b**, **11a**–**c** and **13**)

The 2-(piperidin-4-yl)oxazole (**2**, 1 mmol) and the corresponding intermediate **4a**–**i**, **7a**,**b**, **10a**–**c**, or **12** (1 mmol) in 15 mL of acetone were treated with 1.5 mmol of potassium carbonate as a base and 0.1 mmol of potassium iodide as a catalyst. Using a mixture of CHCl_3_ (1 mL) and MeOH (5 drops) as the developing system, the reaction mixture was refluxed while being stirred for 4 h. The reaction’s progress was tracked using TLC. The related target compounds **5a**–**i**, **8a**,**b**, **11a**–**c**, and **13** were obtained as crystalline powders by collecting the precipitated solid by filtration, washing it with water, crystallizing it from ethanol, and then drying it with diethyl ether under reduced pressure.

***2-(4-(Benzo[d]oxazol-2-yl)piperidin-1-yl)-N-phenylacetamide (5a)***

Beige solid (85%); R_f_ = 0.20; m.p. 140–142 °C; ^1^H NMR *δ*: 9.68 (s, 1H, CONH), 7.67–7.60 (m, 4H, Aromatic protons), 7.41–7.38 (m, 1H, Aromatic proton), 7.31–7.30 (m, 1H, Aromatic proton), 7.26 (t, *J* = 7.6 Hz, 2H, Aromatic protons), 7.01 (t, *J* = 7.2 Hz, 1H, Aromatic proton), 3.11 (s, 2H, CH_2_CO), 3.02–2.96 (m, 1H, CH of piperidine), 2.90–2.87 (m, 2H, NCH_2_ of piperidine), 2.35–2.30 (m, 2H, NCH_2_ of piperidine), 2.08–2.04 (m, 2H, CH_2_ of piperidine), 1.96–1.89 (m, 2H, CH_2_ of piperidine); ^13^C NMR *δ*: 169.4 (C2 of benzoxazole), 168.9 (CH_2_**CO**), 150.5, 141.2, 139.1, 129.4, 129.1, 125.7, 125.2, 124.7, 123.9, 120.0, 119.9, 111.1, 62.5 (**CH_2_**CO), 52.9 (N**(CH_2_)_2_** of piperidine), 35.1 (**CH**(CH_2_)_2_ of piperidine), 29.6 (CH**(CH_2_)_2_** of piperidine); Anal. Calcd. for C_20_H_21_N_3_O_2_ (335.16 g/mol): C, 71.62; H, 6.31; N, 12.53; Found C, 71.40; H, 6.29; N, 12.49%.

***2-(4-(Benzo[d]oxazol-2-yl)piperidin-1-yl)-N-(3-(trifluoromethyl)phenyl)acetamide (5b)***

White solid (80%); R_f_ = 0.29; m.p. 110–112 °C; ^1^H NMR *δ*: 10.02 (s, 1H, CONH), 8.12 (s, 1H, Aromatic proton), 7.88 (d, *J* = 8.2 Hz, 1H, Aromatic proton), 7.64 (dd, *J* = 14.1, 8.2 Hz, 2H, Aromatic protons), 7.49 (t, *J* = 8.2 Hz, 1H, Aromatic proton), 7.35 (d, *J* = 8.2 Hz, 1H, Aromatic proton), 7.32–7.27 (m, 2H, Aromatic protons), 3.15 (s, 2H, CH_2_CO), 3.00–2.94 (m, 1H, CH of piperidine), 2.88 (d, *J* = 8.0 Hz, 2H, NCH_2_ of piperidine), 2.31 (t, *J* = 8.0 Hz, 2H, NCH_2_ of piperidine), 2.07–2.03 (m, 2H, CH_2_ of piperidine), 1.98–1.90 (m, 2H, CH_2_ of piperidine); ^13^C NMR *δ*: 169.2 (C2 of benzoxazole), 168.9 (CH_2_**CO**), 150.1, 140.7, 139.4, 129.8, 129.4 (q, ^2^*J*_CF_ = 31.4 Hz), 126.4 (q, ^1^*J*_CF_ = 273.6 Hz), 124.7, 123.1, 119.7, 119.4, 115.7, 115.7, 110.6, 62.0 (**CH_2_**CO), 52.4 (N**(CH_2_)_2_** of piperidine), 34.6 (**CH**(CH_2_)_2_ of piperidine), 29.0 (CH**(CH_2_)_2_** of piperidine); Anal. Calcd. for C_21_H_20_F_3_N_3_O_2_ (403.15 g/mol): C, 62.53; H, 5.00; N, 10.42; Found C, 62.79; H, 5.01; N, 10.38%.

***2-(4-(Benzo[d]oxazol-2-yl)piperidin-1-yl)-N-(4-fluorophenyl)acetamide (5c)***

White solid (75%); R_f_ = 0.31; m.p. 170–172 °C; ^1^H NMR *δ*: 9.75 (s, 1H, CONH), 7.67–7.62 (m, 4H, Aromatic protons), 7.33–7.28 (m, 2H, Aromatic protons), 7.10 (t, *J* = 10.0 Hz, 2H, Aromatic protons), 3.11 (s, 2H, CH_2_CO), 3.01–2.95 (m, 1H, CH of piperidine), 2.90–2.86 (m, 2H, NCH_2_ of piperidine), 2.34–2.28 (m, 2H, NCH_2_ of piperidine), 2.07–2.04 (m, 2H, CH_2_ of piperidine), 1.97–1.89 (m, 2H, CH_2_ of piperidine); Anal. Calcd. for C_20_H_20_FN_3_O_2_ (353.15 g/mol): C, 67.97; H, 5.70; N, 11.89; Found C, 68.23; H, 5.69; N, 11.84%.

***2-(4-(Benzo[d]oxazol-2-yl)piperidin-1-yl)-N-(4-chlorophenyl)acetamide (5d)***

White solid (82%); R_f_ = 0.36; m.p. 172–174 °C; ^1^H NMR *δ*: 10.10 (s, 1H, CONH), 7.70–7.67 (m, 2H, Aromatic protons), 7.66–7.62 (m, 2H, Aromatic protons), 7.33–7.27 (m, 4H, Aromatic protons), 3.15 (s, 2H, CH_2_CO), 2.99–2.95 (m, 1H, CH of piperidine), 2.88 (d, *J* = 10.0 Hz, 2H, NCH_2_ of piperidine), 2.33–2.29 (m, 2H, NCH_2_ of piperidine), 2.05–2.03 (m, 2H, CH_2_ of piperidine), 1.94–1.87 (m, 2H, CH_2_ of piperidine); ^13^C NMR *δ* 169.4 (C2 of benzoxazole), 169.2 (CH_2_CO), 150.5, 141.2, 138.2, 128.9, 127.3, 125.2, 124.7, 121.5, 119.9, 111.1, 62.3 (CH_2_CO), 52.8 (N**(CH_2_)_2_** of piperidine), 35.1 (**CH**(CH_2_)_2_ of piperidine), 29.6 (CH**(CH_2_)_2_** of piperidine); Anal. Calcd. for C_20_H_20_ClN_3_O_2_ (369.12 g/mol): C, 64.95; H, 5.45; N, 11.36; Found C, 65.17; H, 5.40; N, 11.41%.

***2-(4-(Benzo[d]oxazol-2-yl)piperidin-1-yl)-N-(4-bromophenyl)acetamide (5e)***

White solid (83%); R_f_ = 0.38; m.p. 144–146 °C; ^1^H NMR *δ*: 9.83 (s, 1H, CONH), 7.67–7.60 (m, 4H, Aromatic protons), 7.46–7.43 (m, 2H, Aromatic protons), 7.34–7.28 (m, 2H, Aromatic protons), 3.12 (s, 2H, CH_2_CO), 3.01–2.95 (m, 1H, CH of piperidine), 2.89–2.86 (m, 2H, NCH_2_ of piperidine), 2.34–2.29 (m, 2H, NCH_2_ of piperidine), 2.07–2.04 (m, 2H, CH_2_ of piperidine), 1.96–1.88 (m, 2H, CH_2_ of piperidine); ^13^C NMR *δ*: 169.4 (C2 of benzoxazole), 169.1 (CH_2_**CO**), 150.6, 141.3, 138.5, 131.9, 127.8, 125.2, 124.7, 122.0, 119.9, 115.5, 111.0, 62.5 (**CH_2_**CO), 52.9 (N**(CH_2_)_2_** of piperidine), 35.1 (**CH**(CH_2_)_2_ of piperidine), 29.6 (CH**(CH_2_)_2_** of piperidine); Anal. Calcd. for C_20_H_20_BrN_3_O_2_ (413.07 g/mol): C, 57.98; H, 4.87; N, 10.14; Found C, 58.25; H, 4.86; N, 10.19%.

***2-(4-(Benzo[d]oxazol-2-yl)piperidin-1-yl)-N-(p-tolyl)acetamide (5f)***

Beige solid (75%); R_f_ = 0.33; m.p. 180–182 °C; ^1^H NMR *δ*: 9.59 (s, 1H, CONH), 7.67–7.62 (m, 2H, Aromatic protons), 7.49 (d, *J* = 10.0 Hz, 2H, Aromatic protons), 7.33–7.27 (m, 2H, Aromatic protons), 7.06 (d, *J* = 8.5 Hz, 2H, Aromatic protons), 3.09 (s, 2H, CH_2_CO), 2.99–2.95 (m, 1H, CH of piperidine), 2.87 (d, *J* = 10.0 Hz, 2H, NCH_2_ of piperidine), 2.30 (t, *J* = 10.0 Hz, 2H, NCH_2_ of piperidine), 2.20 (s, 3H, CH_3_), 2.06–2.04 (m, 2H, CH_2_ of piperidine), 1.95–1.88 (m, 2H, CH_2_ of piperidine); ^13^C NMR *δ*: 169.4 (C2 of benzoxazole), 168.7 (CH_2_**CO**), 150.5, 141.2, 136.5, 132.8, 129.5, 125.2, 124.7, 120.0, 119.9, 111.1, 62.5 (**CH_2_**CO), 52.9 (N**(CH_2_)_2_** of piperidine), 35.1 (**CH**(CH_2_)_2_ of piperidine), 29.6 (CH**(CH_2_)_2_** of piperidine), 20.9 (CH_3_); Anal. Calcd. for C_21_H_23_N_3_O_2_ (349.17 g/mol): C, 72.18; H, 6.63; N, 12.03; Found C, 72.21; H, 6.65; N, 12.01%.

***2-(4-(Benzo[d]oxazol-2-yl)piperidin-1-yl)-N-(4-methoxyphenyl)acetamide (5g)***

Semon solid (89%); R_f_ = 0.40; m.p. 140–142 °C; ^1^H NMR *δ*: 9.55 (s, 1H, CONH), 7.67–7.63 (m, 2H, Aromatic protons), 7.52–7.50 (m, 2H, Aromatic protons), 7.34–7.28 (m, 2H, Aromatic protons), 6.84–6.82 (m, 2H, Aromatic protons), 3.67 (s, 3H, OCH_3_), 3.08 (s, 2H, CH_2_CO), 3.01–2.97 (m, 1H, CH of piperidine), 2.88 (d, *J* = 10.0 Hz, 2H, CH_2_ of piperidine), 2.33–2.29 (m, 2H, CH_2_ of piperidine), 2.07–2.04 (d, *J* = 6.0 Hz, 2H, CH_2_ of piperidine), 1.97–1.89 (m, 2H, CH_2_ of piperidine); ^13^C NMR *δ*: 169.4 (C2 of benzoxazole), 168.4 (CH_2_**CO**), 155.8, 150.5, 141.2, 132.2, 125.2, 124.8, 122.0, 121.6, 119.9, 114.2, 111.1, 62.4 (**CH_2_**CO), 55.6 (OCH_3_), 52.9 (N**(CH_2_)_2_** of piperidine), 35.1 (**CH**(CH_2_)_2_ of piperidine), 29.6 (CH**(CH_2_)_2_** of piperidine); Anal. Calcd. for C_21_H_23_N_3_O_3_ (365.17 g/mol): C, 69.02; H, 6.34; N, 11.50; Found C, 68.81; H, 6.35; N, 11.47%.

***2-(4-(Benzo[d]oxazol-2-yl)piperidin-1-yl)-N-(3-chloro-4-fluorophenyl)acetamide (5h)***

White solid (88%); R_f_ = 0.31; m.p. 152–154 °C; ^1^H NMR *δ*: 9.89 (s, 1H, CONH), 7.95 (dd, *J* = 6.9, 2.7 Hz, 1H, Aromatic proton), 7.67–7.62 (m, 2H, Aromatic protons), 7.60–7.57 (m, 1H, Aromatic proton), 7.34–7.28 (m, 3H, Aromatic protons), 3.12 (s, 2H, CH_2_CO), 3.01–2.95 (m, 1H, CH of piperidine), 2.88–2.86 (m, 2H, NCH_2_ of piperidine), 2.33–2.28 (m, 2H, NCH_2_ of piperidine), 2.07–2.03 (m, 2H, CH_2_ of piperidine), 1.98–1.90 (m, 2H, CH_2_ of piperidine); ^13^C NMR *δ*: 169.4 (C2 of benzoxazole), 169.3 (CO), 153.6 (d, ^1^*J*_CF_ = 243.3 Hz), 152.7, 150.5, 141.2, 136.3 (d, ^4^*J*_CF_ = 3.0 Hz), 125.2, 124.7, 121.5, 120.4 (d, ^3^*J*_CF_ = 6.9 Hz), 120.4, 119.9, 119.5, 119.4, 117.2 (d, ^2^*J*_CF_ = 21.7 Hz), 117.1, 111.1, 62.4 (**CH_2_**CO), 52.9 (N**(CH_2_)_2_** of piperidine), 35.1 (**CH**(CH_2_)_2_ of piperidine), 29.5 (CH**(CH_2_)_2_** of piperidine); Anal. Calcd. for C_20_H_19_ClFN_3_O_2_ (387.11 g/mol): C, 61.94; H, 4.94; N, 10.83; Found C, 61.99; H, 4.95; N, 10.87%.

***2-(4-(Benzo[d]oxazol-2-yl)piperidin-1-yl)-N-(4-fluoro-3-(trifluoromethyl)phenyl)acetamide (5i)***

White solid (87%); R_f_ = 0.35; m.p. 138–140 °C; ^1^H NMR *δ*: 10.05 (s, 1H, CONH), 8.14–8.12 (dd, m, 1H, Aromatic proton), 7.96–7.93 (m, 1H, Aromatic proton), 7.66–7.61 (m, 2H, Aromatic proton), 7.42 (t, *J* = 10.0 Hz, 1H, Aromatic proton), 7.32–7.27 (m, 2H, Aromatic proton), 3.14 (s, 2H, CH_2_CO), 2.99–2.95 (m, 1H, CH of piperidine), 2.88 (d, *J* = 10.0 Hz, 2H, NCH_2_ of piperidine), 2.31 (t, *J* = 10.0 Hz, 2H, NCH_2_ of piperidine), 2.07–2.03 (m, 2H, CH_2_ of piperidine), 1.99–1.91 (m, 2H, CH_2_ of piperidine); ^13^C NMR *δ*: 169.5 (C2 of benzoxazole), 169.4 (CO), 154.9 (d, *J*_CF_ = 250.1 Hz), 150.5, 141.2, 135.9, 135.9, 126.1 (d, *J*_CF_ = 7.9 Hz), 125.0 (d, *J*_CF_ = 272.4 Hz), 121.9, 119.9, 118.2 (d, *J*_CF_ = 5.0 Hz), 118.2, 118.1, 118.1, 117.9 (d, *J*_CF_ = 21.2 Hz), 111.0, 62.4 (**CH_2_**CO), 52.9 (N**(CH_2_)_2_** of piperidine), 35.1 (**CH**(CH_2_)_2_ of piperidine), 29.5 (CH**(CH_2_)_2_** of piperidine); Anal. Calcd. for C_21_H_19_F_4_N_3_O_2_ (421.14 g/mol): C, 59.86; H, 4.54; N, 9.97; Found C, 59.89; H, 4.53; N, 9.95%.

***2-(4-(Benzo[d]oxazol-2-yl)piperidin-1-yl)-N-benzylacetamide (8a)***

White solid (91%); R_f_ = 0.28; m.p. 114–116 °C; ^1^H NMR *δ*: 8.31 (t, *J* = 7.5 Hz, 1H, CONH), 7.66–7.61 (m, 2H, Aromatic protons), 7.33–7.25 (m, 4H, Aromatic protons), 7.23–7.16 (m, 3H, Aromatic protons), 4.27 (d, *J* = 7.5 Hz, 2H, NH**CH_2_**), 2.96 (s, 2H, CH_2_CO), 2.95–2.92 (m, 1H, CH of piperidine), 2.82 (d, *J* = 10.0 Hz, 2H, NCH_2_ of piperidine), 2.23 (t, *J* = 10.0 Hz, 2H, NCH_2_ of piperidine), 2.00–2.00 (m, 2H, CH_2_ of piperidine), 1.93–1.85 (m, 2H, CH_2_ of piperidine); ^13^C NMR *δ* 170.0 (C2 of benzoxazole), 169.4 (CH_2_**CO**), 150.5, 141.2, 140.3, 128.7, 127.6, 127.1, 125.2, 124.7, 119.9, 111.0, 62.0 (**CH_2_**CO), 53.1 (N**(CH_2_)_2_** of piperidine), 42.3 (**CH_2_**-NH), 35.1 (**CH**(CH_2_)_2_ of piperidine), 29.6 (CH**(CH_2_)_2_** of piperidine); Anal. Calcd. for C_21_H_23_N_3_O_2_ (349.17 g/mol): C, 72.18; H, 6.63; N, 12.03; Found C, 72.35; H, 6.64; N, 12.08%.

***2-(4-(Benzo[d]oxazol-2-yl)piperidin-1-yl)-N-phenethylacetamide (8b)***

Semon solid (89%); R_f_ = 0.36; m.p. 108–110 °C; ^1^H NMR *δ*: 7.71 (t, *J* = 5.0 Hz, 1H, CONH), 7.67–7.63 (m, 2H, Aromatic protons), 7.33–7.28 (m, 2H, Aromatic protons), 7.24 (t, *J* = 10.0 Hz, 2H, Aromatic protons), 7.17–7.12 (m, 3H, Aromatic protons), 3.33–3.32 (m, 2H, NHCH_2_CH_2_), 2.94–2.90 (m, 1H, CH of piperidine), 2.84 (s, 2H, CH_2_CO), 2.72–2.67 (m, 4H, NH-CH_2_-CH_2_ & NCH_2_ of piperidine), 2.13 (t, *J* = 10.0 Hz, 2H, NCH_2_ of piperidine), 1.98–1.94 (m, 2H, CH_2_ of piperidine), 1.85–1.77 (m, 2H, CH_2_ of piperidine); ^13^C NMR *δ*: 169.7 (C2 of benzoxazole), 169.4 (CH_2_**CO**), 150.5, 141.2, 139.9, 129.1, 128.8, 126.6, 125.2, 124.7, 119.9, 111.1, 62.0 (**CH_2_**CO), 53.0 (N**(CH_2_)_2_** of piperidine), 40.0 (**CH_2_**NH), 35.5 (**CH_2_**-CH_2_-NH), 35.0 (**CH**(CH_2_)_2_ of piperidine), 29.7 (CH**(CH_2_)_2_** of piperidine); Anal. Calcd. for C_22_H_25_N_3_O_2_ (363.19 g/mol): C, 72.70; H, 6.93; N, 11.56; Found C, 72.49; H, 6.92; N, 11.59%.

***2-(4-(Benzo[d]oxazol-2-yl)piperidin-1-yl)-1-phenylethan-1-one (11a)***

Peach solid (84%); R_f_ = 0.21; m.p. 120–122 °C; ^1^H NMR *δ*: 7.96 (dd, *J* = 10.0, 5.0, 2H, Aromatic protons), 7.66–7.57 (m, 3H, Aromatic protons), 7.49–7.46 (m, 2H, Aromatic protons), 7.31–7.29 (m, 2H, Aromatic protons), 3.83 (s, 2H, CH_2_CO), 3.00–2.95 (m, 1H, CH of piperidine), 2.92–2.88 (m, 2H, NCH_2_ of piperidine), 2.33–2.28 (m, 2H, NCH_2_ of piperidine), 2.05–2.01 (m, 2H, CH_2_ of piperidine), 1.83–1.75 (m, 2H, CH_2_ of piperidine); ^13^C NMR *δ*: 197.7 (CO), 169.4 (C2 of benzoxazole), 150.5, 141.2, 136.4, 133.7, 129.7, 129.1, 129.0, 128.6, 125.2, 124.7, 119.9, 111.1, 64.5 (**CH_2_**CO), 52.7 (N**(CH_2_)_2_** of piperidine), 35.2 (**CH**(CH_2_)_2_ of piperidine), 29.7 (CH**(CH_2_)_2_** of piperidine); Anal. Calcd. for C_20_H_20_N_2_O_2_ (320.15 g/mol): C, 74.98; H, 6.29; N, 8.74; Found C, 74.95; H, 6.30; N, 8.79%.

***2-(4-(Benzo[d]oxazol-2-yl)piperidin-1-yl)-1-(4-fluorophenyl)ethan-1-one (11b)***

Cotton candy solid (87%); R_f_ = 0.23; m.p. 130–132 °C; ^1^H NMR ***δ***: 8.08–8.00 (m, 2H, Aromatic protons), 7.4–7.95 (m, 2H, Aromatic protons), 7.31–7.25 (m, 4H, Aromatic protons), 3.79 (s, 2H, CH_2_CO), 2.98–2.92 (m, 1H, CH of piperidine), 2.89–2.85 (m, 2H, NCH_2_ of piperidine), 2.30–2.25 (m, 2H, NCH_2_ of piperidine), 2.05–1.96 (m, 2H, CH_2_ of piperidine), 1.82–1.74 (m, 2H, CH_2_ of piperidine); ^13^C NMR *δ*: 196.3 (CO), 169.4 (C2 of benzoxazole), 165.4 (d, ^1^*J*_CF_ = 252.2 Hz), 150.5, 141.1, 132.9 (d, ^4^*J*_CF_ = 2.7 Hz), 131.6 (d, ^3^*J*_CF_ = 9.4 Hz), 125.2, 124.7, 119.8, 116.0 (d, ^2^*J*_CF_ = 21.9 Hz), 111.0, 64.5 (**CH_2_**CO), 52.7 (N**(CH_2_)_2_** of piperidine), 35.2 (**CH**(CH_2_)_2_ of piperidine), 29.6 (CH**(CH_2_)_2_** of piperidine); Anal. Calcd. for C_20_H_19_FN_2_O_2_ (338.14 g/mol): C, 70.99; H, 5.66; N, 8.28; Found C, 71.18; H, 5.68; N, 8.31%.

***2-(4-(Benzo[d]oxazol-2-yl)piperidin-1-yl)-1-(4-chlorophenyl)ethan-1-one (11c)***

Beige solid (75%); R_f_ = 0.22; m.p. 154–156 °C; ^1^H NMR *δ*: 7.98 (d, *J* = 8.0 Hz, 2H, Aromatic protons), 7.65–7.61 (m, 2H, Aromatic protons), 7.54 (d, *J* = 10.0 Hz, 2H, Aromatic protons), 7.31–7.27 (m, 2H, Aromatic protons), 3.80 (s, 2H, CH_2_CO), 2.99–2.93 (m, 1H, CH of piperidine), 2.89–2.85 (m, 2H, NCH_2_ of piperidine), 2.28 (t, *J* = 10.0 Hz, 2H, NCH_2_ of piperidine), 2.03–2.00 (m, 2H, CH_2_ of piperidine), 1.82–1.74 (m, 2H, CH_2_ of piperidine); ^13^C NMR *δ*: 196.8 (CO), 169.3 (C2 of benzoxazole), 150.5, 141.2, 138.6, 135.0, 131.6, 130.6, 129.2, 129.0, 125.2, 124.7, 119.9, 111.1, 64.7 (**CH_2_**CO), 52.7 (N**(CH_2_)_2_** of piperidine), 35.2 (**CH**(CH_2_)_2_ of piperidine), 29.7 (CH**(CH_2_)_2_** of piperidine); Anal. Calcd. for C_20_H_19_ClN_2_O_2_ (354.11 g/mol): C, 67.70; H, 5.40; N, 7.89; Found C, 67.94; H, 5.42; N, 7.94%.

***2-(1-Benzylpiperidin-4-yl)benzo[d]oxazole (13)***

Pale peach solid (78%); R_f_ = 0.28; m.p. 188–190 °C; ^1^H NMR *δ:* 7.66–7.61 (m, 2H, Aromatic protons), 7.32–7.26 (m, 6H, Aromatic protons), 7.22–7.18 (m, 1H, Aromatic proton), 3.45 (s, 2H, NCH_2_), 2.98–2.92 (m, 1H, CH of piperidine), 2.82–2.78 (m, 2H, NCH_2_ of piperidine), 2.11–2.01 (m, 4H, NCH_2_ and CH_2_ of piperidine), 1.82–1.74 (m, 2H, CH_2_ of piperidine).; Anal. Calcd. for C_19_H_20_N_2_O (292.15 g/mol): C, 78.05; H, 6.90; N, 9.58; Found C, 77.89; H, 6.91; N, 9.60%.

### 3.2. Biology Protocols

#### 3.2.1. VEGFR-2 Inhibition Assay

Using a VEGFR-2 Kinase Assay Kit (#40325; BPS Bioscience, San Diego, CA, USA), the drugs’ inhibitory efficacy against VEGFR-2 tyrosine kinase was assessed. Kinase-Glo MAX reagent (Promega, Madison, WI, USA) was added after serial dilutions (0.001–3 µM in DMSO) were evaluated and incubated for 45 min at room temperature with the buffer system [Poly (Glu:Tyr, 4:1) kinase substrate, ATP (10 µM final concentration), and kinase assay buffer]. A BioTek Synergy 2 microplate reader (BioTek Instruments, Winooski, VT, USA) was used to record luminescence in order to calculate IC_50_ values. For background correction, blank controls—assay buffer rather than compounds—were employed [29].

#### 3.2.2. c-Met Inhibition Assay

The c-Met Kinase Assay Kit (#79559; BPS Bioscience) was used to evaluate compound **11b**’s inhibitory efficacy against c-Met kinase. Buffer system contained Poly (Glu:Tyr, 4:1) kinase substrate, ATP (10 µM final concentration), and kinase assay buffer to a final volume of 50 mL. The reaction mixture was incubated at 30 °C for 45 min, and then Kinase-Glo MAX (Promega #V6071) reagent (50 mL) was added to each well of the reaction plate. A BioTekTM Synergy 2 microplate reader was used to monitor chemiluminescence and test serial dilutions (0.003–10 µM in DMSO). The final concentration of DMSO did not exceed 1%. The concentration–response curves were used to obtain the IC_50_ values. Every assay was carried out three times [30].

#### 3.2.3. In Vitro Antiproliferative Assay

Using normal MCF-10A cells as a control, the MTT test was used to evaluate the antiproliferative activity of drugs against human breast (MCF-7), lung (A549), and prostate (PC-3) cancer cell lines [31]. Seeded in 96-well plates, cells (2.1–2.3 × 10^4^/well) were cultivated at 37 °C in 5% CO_2_ in RPMI media supplemented with 10% FCS, antibiotics (penicillin-streptomycin mixture), and bovine insulin (for MCF-7). MCF10A cells were cultured in freshly prepared DMEM/Ham’s F12 (1:1) media immediately supplemented with EGF ‘0.02 g/mL’, hydrocortisone ‘0.5 g/mL’, cholera toxin ‘0.1 g/mL’, horse serum ‘5%’, penicillin-streptomycin mixture ‘1%’ and 0.01 mg/mL bovine insulin. Compound stock solutions were made in DMSO and diluted one at a time to eight different doses (0.1–300 µM). MTT (0.5 μg/μL) was added after 72 h of treatment, and the mixture was incubated for 4 h before the formazan crystals were dissolved using DMSO:isopropanol (1:1). A microplate reader was used to measure absorbance at 590 nm. Three of each experiment were conducted. Finally, IC_50_ values were determined by non-linear regression of dose–response curves using GraphPad Prism 8.

#### 3.2.4. Cell Cycle Analysis

Propidium iodide (PI) staining was used to examine the cell cycle distribution when MCF-7 cells were exposed to compound **11b** at the IC_50_ concentration for 72 h [32]. Cells were fixed in 70% ethanol at 4 °C, rinsed with PBS, stained with PI (0.02 mg/mL) and RNase A (0.1 mg/mL), and then left to incubate for 30 min at 37 °C in the dark. An Epics XL-MCLTM flow cytometer (Beckman Coulter, Brea, CA, USA) was used to determine the DNA content and quantify the cell populations at the sub-G_1_, G_1_, S, and G_2_/M phases.

#### 3.2.5. Detection of Apoptosis

An Annexin V-FITC/PI kit was used to measure apoptosis in MCF-7 cells in accordance with established procedures [29]. Compound **11b** (4.30 µM) was applied to cells for 72 h, followed by PBS washing, 70% ethanol fixation, and 20 min of dark staining with Annexin V-FITC/PI (BioVision, Milpitas, CA, USA). Using an ACEA Novocyte^TM^ flow cytometer (ACEA Biosciences, San Diego, CA, USA), apoptotic populations were measured. The Annexin V-FITC/PI scatter plots demonstrated viable cells in the lower left quadrants (An^−^, PI^−^), early apoptotic cells in the lower right quadrants (An^+^, PI^−^), late apoptotic cells in upper right quadrants (An^+^, PI^+^), and necrotic cells in the upper left quadrants (An^−^, PI^+^), excluding the cell debris at the bottom left corner of SSC A vs. FSC A plots following the manufacturer’s instructions.

#### 3.2.6. Quantitative Reverse Transcription PCR (qRT-PCR)

In MCF-7 cells treated with compound **11b** at IC_50_ concentration, the mRNA levels of BAX, Bcl-2, caspase-8, and caspase-9 were measured by qRT-PCR in comparison to the vehicle control (0.01% DMSO) [33]. The Rotor-Gene Q heat cycler was used to isolate total RNA using the RNeasy Mini Kit (Qiagen, Hilden, Germany), reverse-transcribe it to cDNA using the RevertAid First Strand Synthesis Kit (Thermo Scientific, Waltham, MA, USA), and magnify it using the SYBR^®^ Green Master Mix (Bio-Rad, Hercules, CA, USA). The housekeeping gene was GAPDH. In triplicate, reactions were conducted in 20 μL quantities. Finally, the relative quantification of gene expression was accomplished using the 2^−ΔΔCT^ method, with the specificity of each gene amplification verified at the end of the qPCR reactions through the melt curve analysis of the PCR products, using primers listed in the Supplementary Table S1.

#### 3.2.7. Molecular Modelling

The RCSPDB was used to obtain the c-Met (PDB: 4R1V) [34] and VGFR-2 (PDB: 4ASD) [35] for molecular docking experiments. In the docking investigation, the cocrystal ligands and compounds **11a** and **11b**, putative inhibitors, were employed. AutoDock Vina 1.1.2 was operated for carrying docking [36].

## 4. Conclusions

To summarize, compound **11b** was identified as a highly promising lead structure by the logical design and synthesis of novel dual c-Met/VEGFR-2 inhibitors. With submicromolar IC_50_ values against c-Met and VEGFR-2, the compound exhibited extraordinary dual inhibitory activity, demonstrating its capacity to concurrently disrupt two key signaling pathways associated with breast cancer angiogenesis, proliferation, and metastasis. Compound **11b** exhibited strong antiproliferative activity against MCF-7 cells, in addition to enzymatic inhibition, causing cell cycle arrest at the G_2_/M stages and inducing apoptosis in over half of the treated population. From the standpoint of medicinal chemistry, our results highlight the value of dual kinase inhibition as a successful strategy to overcome adaptive resistance and route redundancy, which are frequently encountered in cancer treatment. Compound **11b**’s structural characteristics provide a useful framework for future optimization of potency and pharmacokinetic properties. The observed relationship between enzyme inhibition and cellular outcomes further supports its potential for translation into advanced preclinical studies. All things considered, this study identifies compound **11b** as a strong dual c-Met/VEGFR-2 inhibitor and a promising chemotherapeutic lead candidate for the treatment of breast cancer, highlighting the need for further research into its in vivo effectiveness, selectivity profile, and potential combination regimens.

## Figures and Tables

**Table 1:** IC_50_ * values of the tested compounds as VEGFR-2 & c-Met kinases inhibition assay.

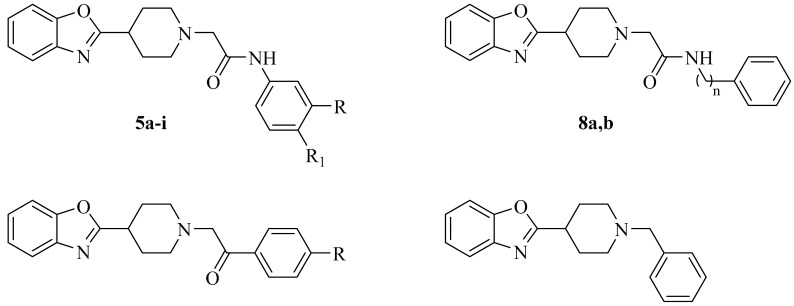					
Code	R	R_1_	*n* **	IC_50_ (μM) (Mean ± SEM)	
				VEGFR-2	c-Met
**5a**	H	H	-	0.145 ± 0.04	1.382 ± 0.51
**5b**	CF_3_	H	-	1.107 ± 0.39	0.513 ± 0.12
**5c**	H	F	-	0.323 ± 0.10	1.250 ± 0.29
**5d**	H	Cl	-	0.672 ± 0.29	0.933 ± 0.08
**5e**	H	Br	-	1.578 ± 0.53	0.983 ± 0.47
**5f**	H	CH_3_	-	0.823 ± 0.10	1.126 ± 0.38
**5g**	H	OCH_3_	-	0.131 ± 0.03	0.970 ± 0.09
**5h**	Cl	F	-	0.152 ± 0.03	1.885 ± 0.30
**5i**	CF_3_	F	-	1.514 ± 0.45	2.210 ± 0.37
**8a**	-	-	1	1.680 ± 0.42	1.108 ± 0.51
**8b**	-	-	2	0.219 ± 0.02	1.490 ± 0.20
**11a**	H	-	-	0.082 ± 0.01	0.280 ± 0.03
**11b**	F	-	-	0.057 ± 0.01	0.181 ± 0.04
**11c**	Cl	-	-	0.625 ± 0.24	0.898 ± 0.16
**13**	-	-	-	0.502 ± 0.11	0.590 ± 0.21
Sorafenib	-	-	-	0.058 ± 0.01	--
Staurosporine	-	-	-	--	0.237 ± 0.01

* Data are presented as mean of the IC_50_ values ± SEM from three different experiments. ** *n* denotes linker length.

**Table 2:** In vitro antiproliferative activities of the most potent compounds against human breast (MCF-7), lung (A549), prostate (PC-3) cancer cell lines, and the normal MCF-10A cells (72 h).

Code	In Vitro Cytotoxicity [IC_50_, µM ± SEM] ^a^				
	MCF-7	A549	PC-3	MCF-10A	MCF-10A/MCF-7 Ratio
**5a**	16.29 ± 2.07	23.60 ± 1.01	16.14 ± 1.13	28.31 ± 1.23	1.74
**5g**	17.23 ± 1.29	9.32 ± 1.26	22.63 ± 1.50	30.35 ± 2.38	1.76
**5h**	14.01 ± 0.94	14.86 ± 0.82	24.89 ± 3.01	27.85 ± 3.29	1.99
**11a**	6.25 ± 0.63	8.33 ± 0.64	15.95 ± 0.58	27.17 ± 2.10	4.35
**11b**	4.30 ± 0.25	6.68 ± 1.08	7.06 ± 1.10	34.25 ± 1.24	7.97
Sorafenib	4.95 ± 0.92	6.32 ± 0.66	6.57 ± 0.61	21.63 ± 2.81	4.37

^a^ Data are presented as the mean of the IC_50_ values ± SEM from three different experiments.

**Table 3:** Effect of **11b** on levels of BAX, Bcl-2, active caspase-9, and p53 genes expression in MCF-7 cells treated for 72 h.

Sample	Gene Expression (Normalized to GAPDH) ^a^				
	BAX	Bcl-2	BAX/Bcl-2 Ratio	Caspase-9	p53
MCF-7 cells	1.00 ± 0.09	1.00 ± 0.04	1.00 ± 0.08	1.00 ± 0.06	1.00 ± 0.08
**11b**/MCF-7	2.48 ± 0.40 *	0.51 ± 0.06 **	4.76 ± 0.26 ***	2.48 ± 0.24 **	4.56 ± 0.71 **

^a^ Values are given as changes from the corresponding control (MCF-7) group, which was set to ‘1’. * *p* < 0.05, ** *p* < 0.01 and *** *p* < 0.001 indicate statistically significant differences from the corresponding control in unpaired *t*-tests.

**Table 4:** Percent activity values of compound **11a** against a small panel of 6 kinases at 10 µM.

Kinase	Kinase Activity (%)	Kinase	Kinase Activity (%)
PDGFRβ	65	BTK	84
RET	99	FLT-3	91
GSK3b	100	Aurora B	96

**Table 5:** Molecular Docking Analysis of the tested compounds **11a** and **11b** against VEGFR-2 TK and c-Met kinase.

Tested Targets	Compounds	RMSD Value (Å)	Docking (Affinity) Score (kcal/mol)
VEGFR-2 TK	Compound **11a**	0.94	−8.57
	Compound **11b**	1.14	−8.62
	Sorafenib	0.15	−9.18
c-Met Kinase	Compound **11a**	1.36	−7.98
	Compound **11b**	1.27	−7.93
	Staurosporine	0.18	−9.27

## Data Availability

The original contributions presented in this study are included in the article/Supplementary Materials. Further inquiries can be directed to the corresponding authors.

## Supplementary Materials

The following supporting information can be downloaded at: https://www.mdpi.com/article/10.3390/ph18121875/s1, Figures S1–S28. NMR spectra of compounds **5a**–**i**, **8a**,**b**, **11a**–**c** and **13**; Table S1. Sequences for primers.
