# Supplementary material for: Discovery of Novel Piperidinyl-Based Benzoxazole Derivatives as Anticancer Agents Targeting VEGFR-2 and c-Met Kinases
Source: Pharmaceuticals (Basel). 2025 Dec 9;18(12):1875. doi: 10.3390/ph18121875 (PMC12735468; doi:10.3390/ph18121875)
Supplement: Supplementary file 1 [file pharmaceuticals-18-01875-s001.zip › pharmaceuticals-4026531-supplementary.pdf]

Supporting Information

Discovery of Novel Piperidinyl-Based Benzoxazole Derivatives as Anticancer Agents Targeting VEGFR-2 and c-Met kinases

Wagdy M. Eldehna [1,*], Zainab M. Elsayed [2], Mohamed R. Elnagar [3,4], Ahmed H. El-Said [5], Taghreed A Majrashi [6], Ahmed T. Negmeldin [7,8,*], Abdulrahman M. Saleh [9], Ranza Elrayess [10,11], Khaled A. Elnahriry [12], Zhi-Long Chen [13], Mohamed Elagawany [14], Haytham O. Tawfik [15,*]

[1]*Department of Pharmaceutical Chemistry, Faculty of Pharmacy, Kafrelsheikh University, P.O. Box 33516, Kafrelsheikh, Egypt.*

[2]*Scientific Research and Innovation Support Unit, Faculty of Pharmacy, Kafrelsheikh University, Kafrelsheikh 33516, Egypt.*

[3]*Department of Pharmacology and Toxicology, Faculty of Pharmacy, Al-Azhar University, Cairo 11823, Egypt.*

[4] *Department of Pharmacology, College of Pharmacy, The Islamic University, Najaf 54001, Iraq.*

[5] *Department of Pharmaceutical Chemistry, Faculty of Pharmacy, Delta University for Science and Technology, International Coastal Road, Gamasa City, Mansoura 11152, Dakahliya, Egypt.*

[6] *Department of Pharmacognosy, College of Pharmacy, King Khalid University, Asir, Saudi Arabia.*

[7] *Department of Pharmaceutical Sciences, College of Pharmacy and Thumbay Research Institute for Precision Medicine, Gulf Medical University, Ajman, United Arab Emirates.*

[8] *Department of Pharmaceutical Organic Chemistry, Faculty of Pharmacy, Cairo University, Cairo, Egypt.*

[9] *Department of Pharmaceutical Chemistry, Faculty of Pharmacy, Cairo University, Kasr El-Aini Street, Cairo, 11562, Egypt.*

[10] *Pharmaceutical Organic Chemistry Department, Faculty of Pharmacy, Suez Canal University, Ismailia 41522, Egypt.*

[11] *Pharmaceutical Organic Chemistry Department, College of Pharmacy, Al-Ayen Iraqi University, AUIQ, An Nasiriyah, Iraq, 64001.*

[12] *Department of Pharmaceutical Sciences, College of Pharmacy, Alfaisal University, Riyadh 11533, Saudi Arabia.*

[13] *Department of Pharmaceutical Science and Technology, Donghua University. Shanghai 201620, China.*

[14] *Department of Pharmaceutical Chemistry, Faculty of Pharmacy, Damanhour University, Damanhour, Buhaira, 22516, Egypt.*

[15] *Department of Pharmaceutical Chemistry, Faculty of Pharmacy, Tanta University, Tanta, 31527, Egypt.*

*Corresponding authors: W. M. Eldehna wagdy2000@gmail.com, A. T. Negmeldin dr.ahmedthabet@gmu.ac.ae and H. O. Tawfik haytham.omar.mahmoud@pharm.tanta.edu.eg.

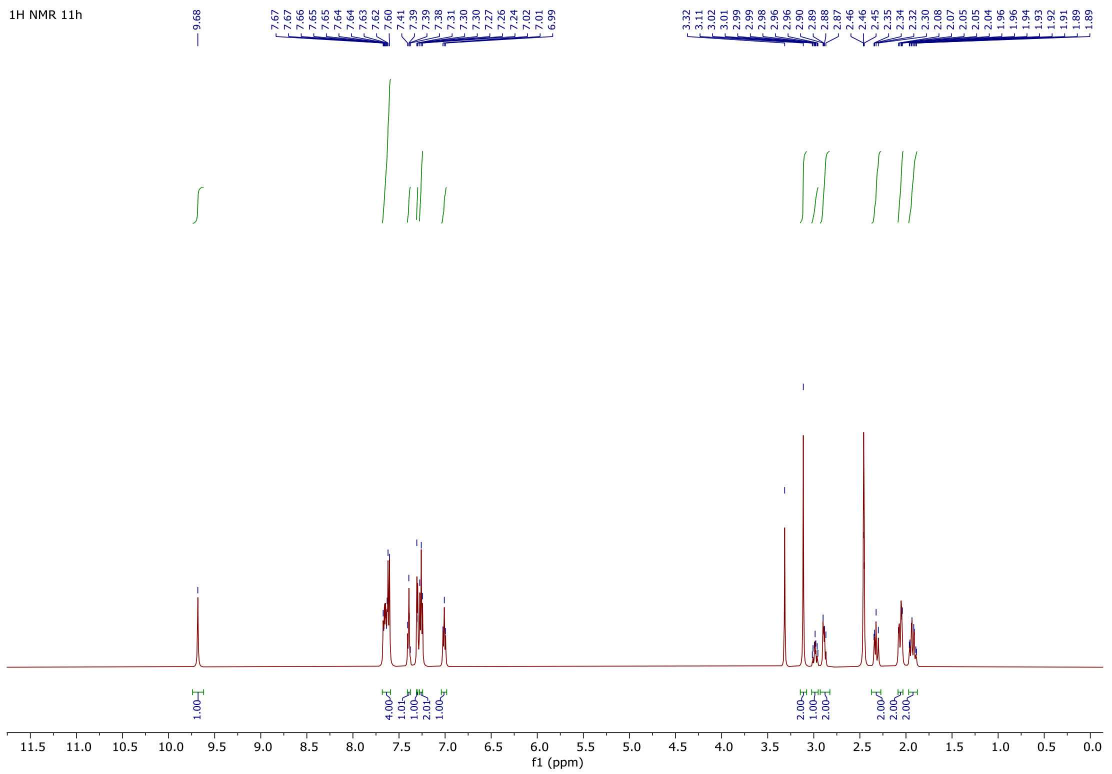

Figure S1. ^{1}H NMR (500 MHz, DMSO-d_6) spectrum of compound **5a**

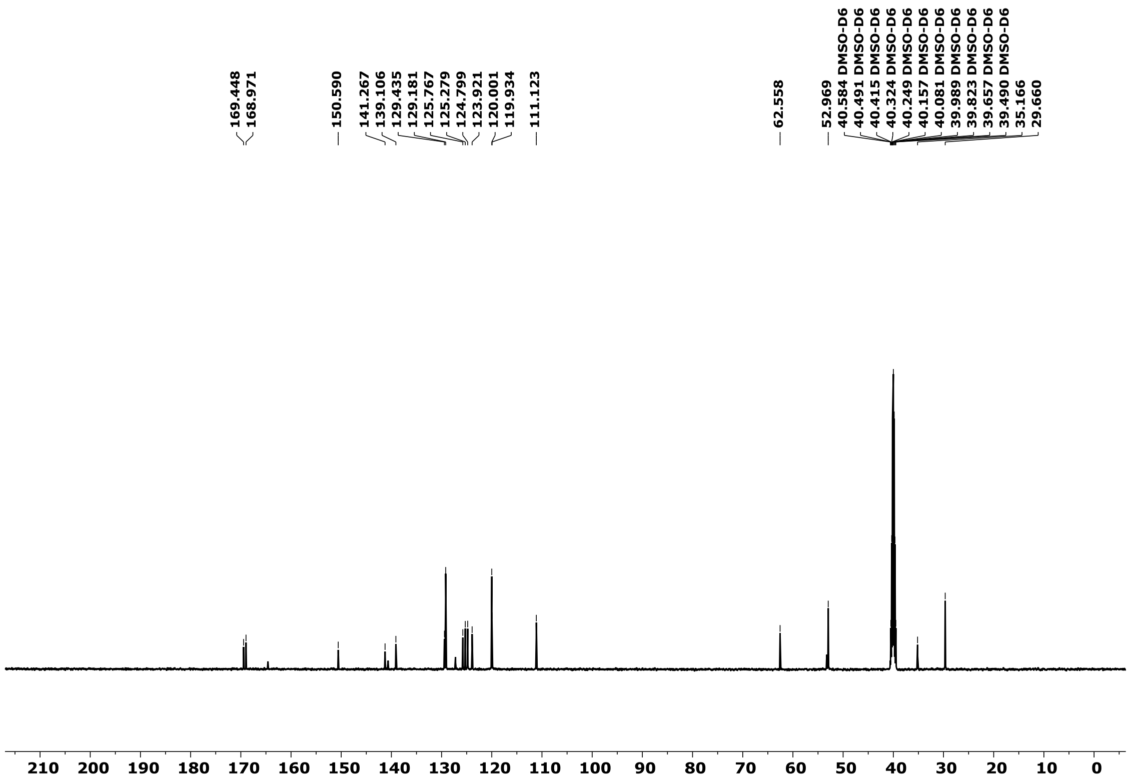

Figure S2. ^{13}C NMR (126 MHz, DMSO-d_6) spectrum of compound **5a**

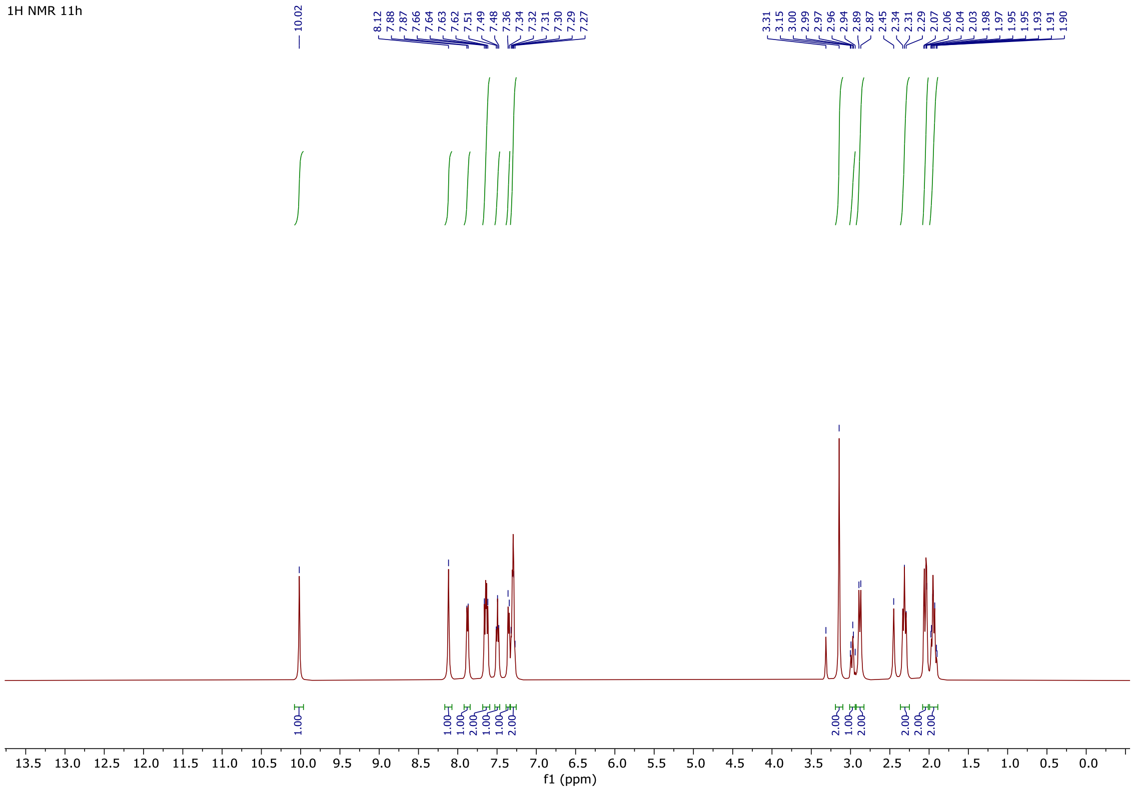

Figure S3. ^{1}H NMR (500 MHz, DMSO-d_6) spectrum of compound **5b**

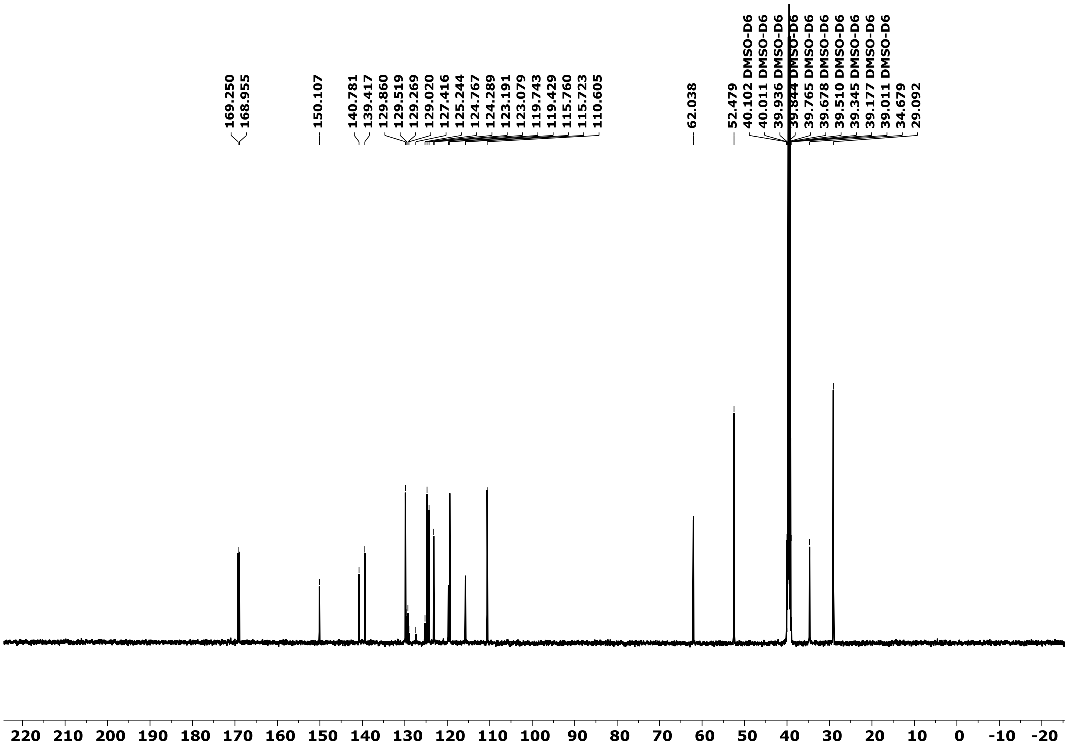

Figure S4. ^{13}C NMR (126 MHz, DMSO-d_6) spectrum of compound **5b**

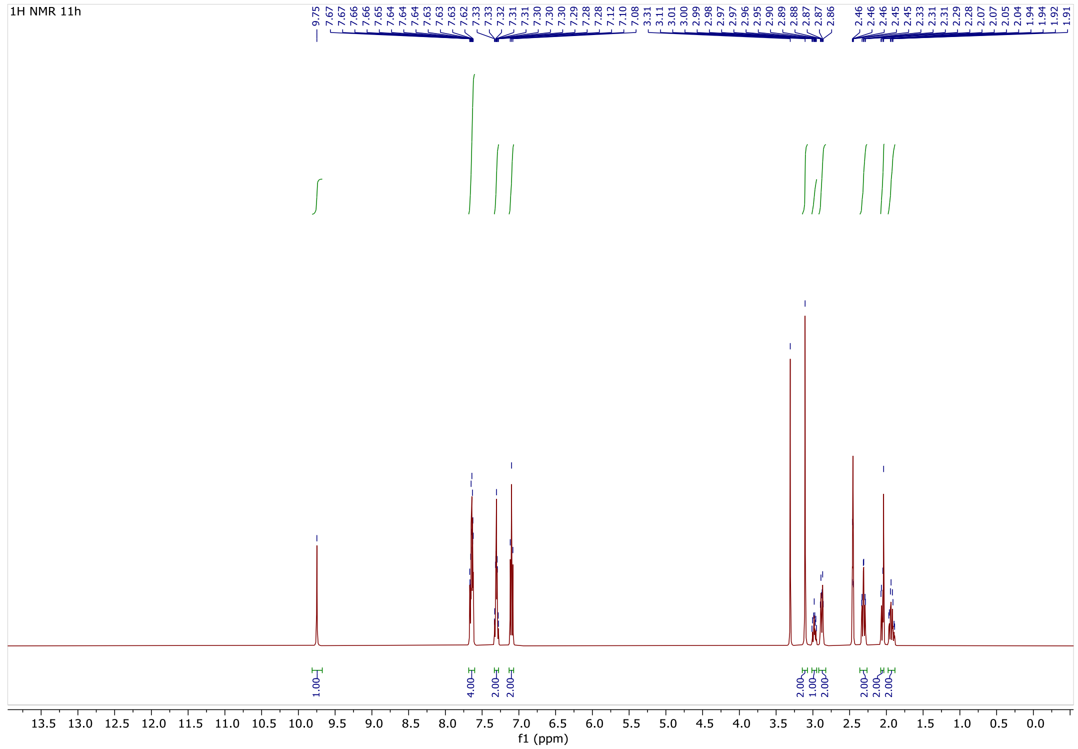

Figure S5. ^{1}H NMR (500 MHz, DMSO-d_6) spectrum of compound **5c**

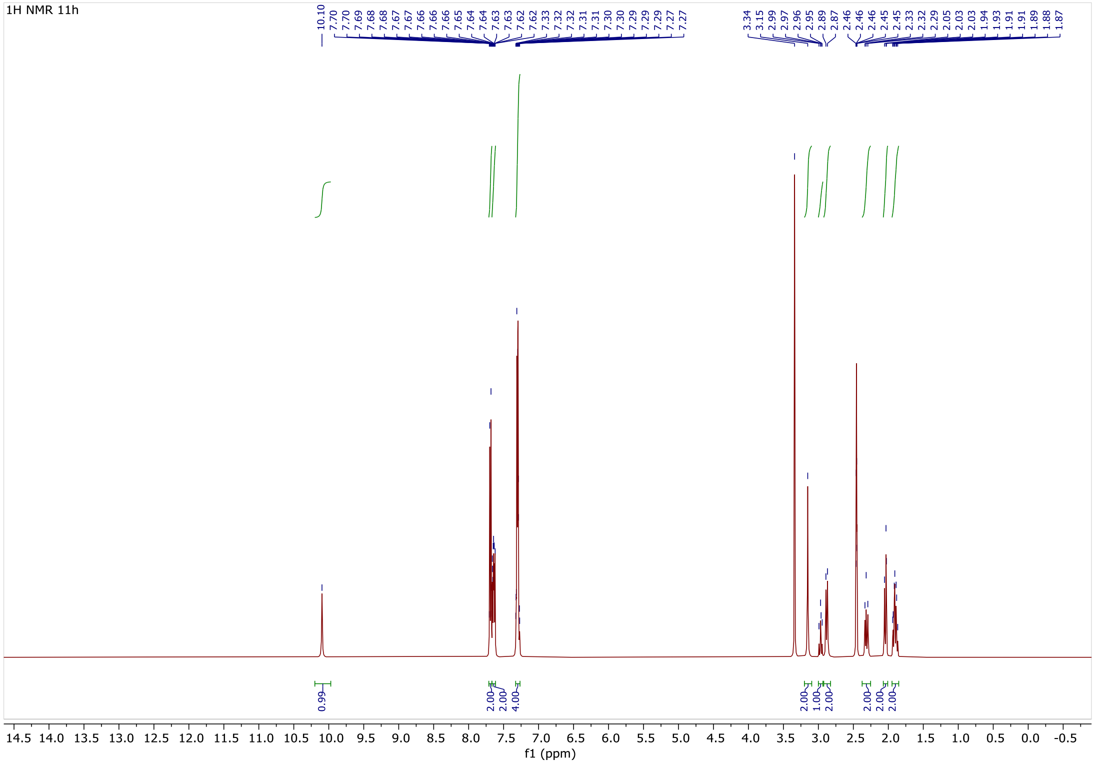

Figure S6. ^{1}H NMR (500 MHz, DMSO-d_6) spectrum of compound **5d**

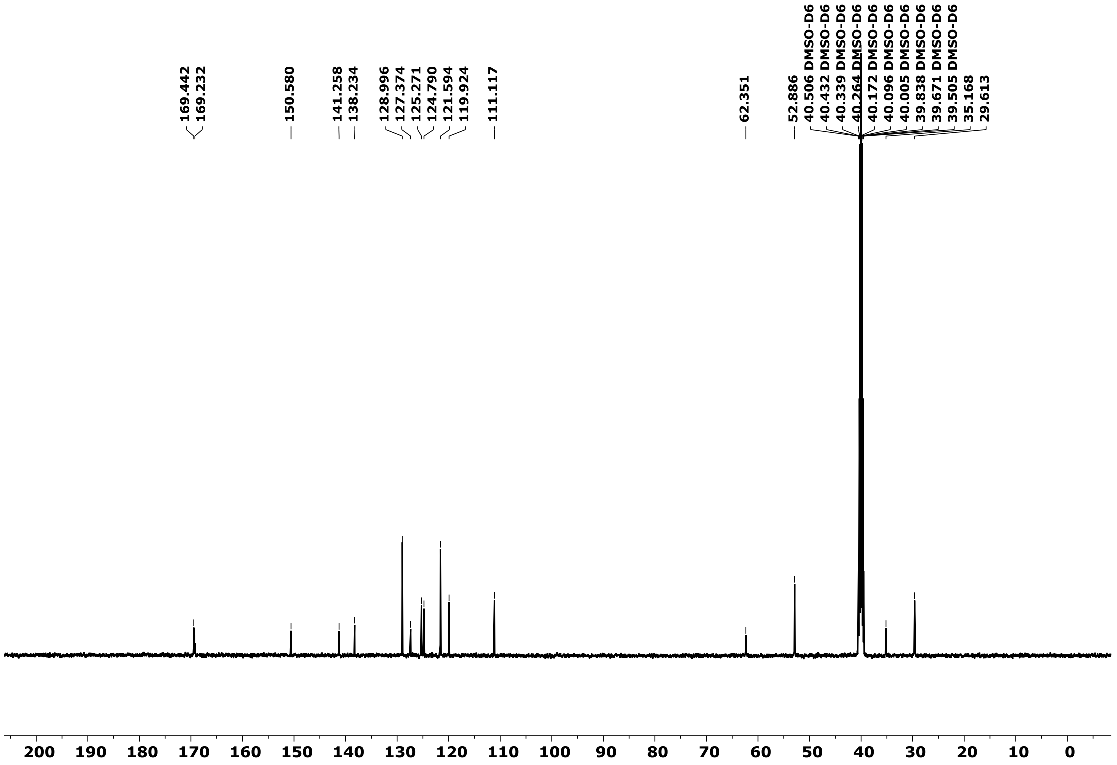

Figure S7. ^{13}C NMR (126 MHz, DMSO-d_6) spectrum of compound **5d**

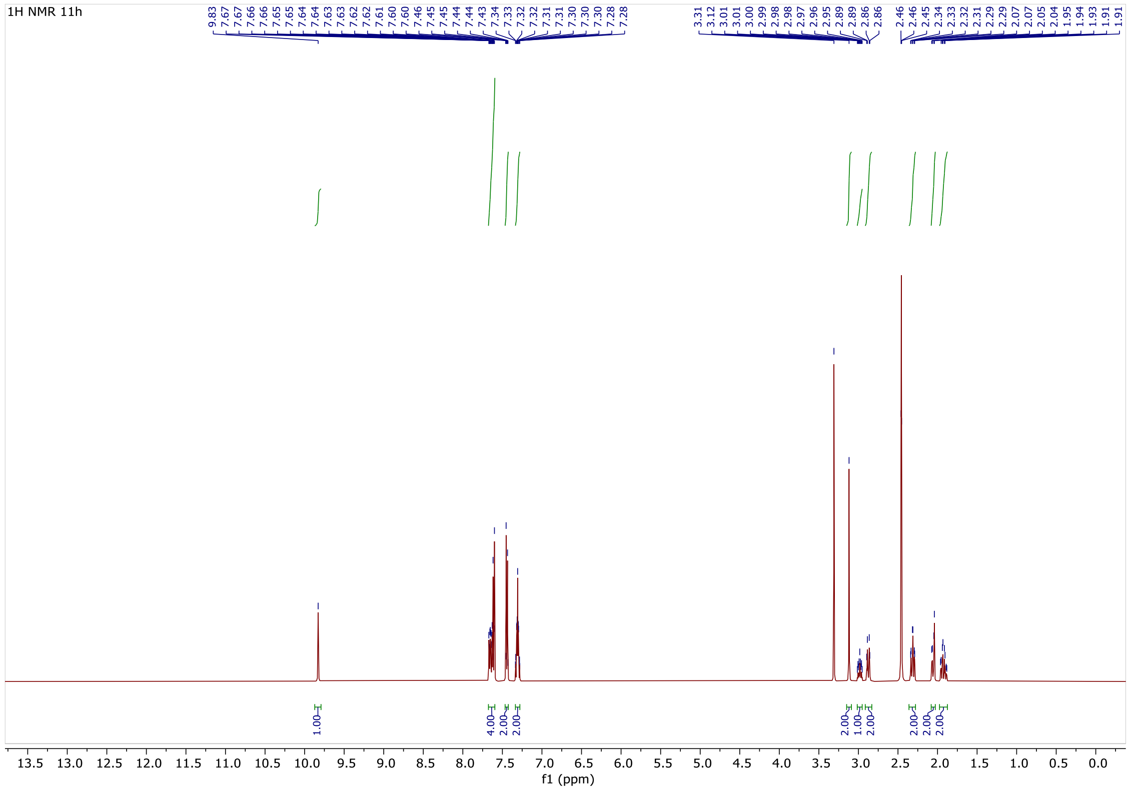

Figure S8. ^{1}H NMR (500 MHz, DMSO-d_6) spectrum of compound **5e**

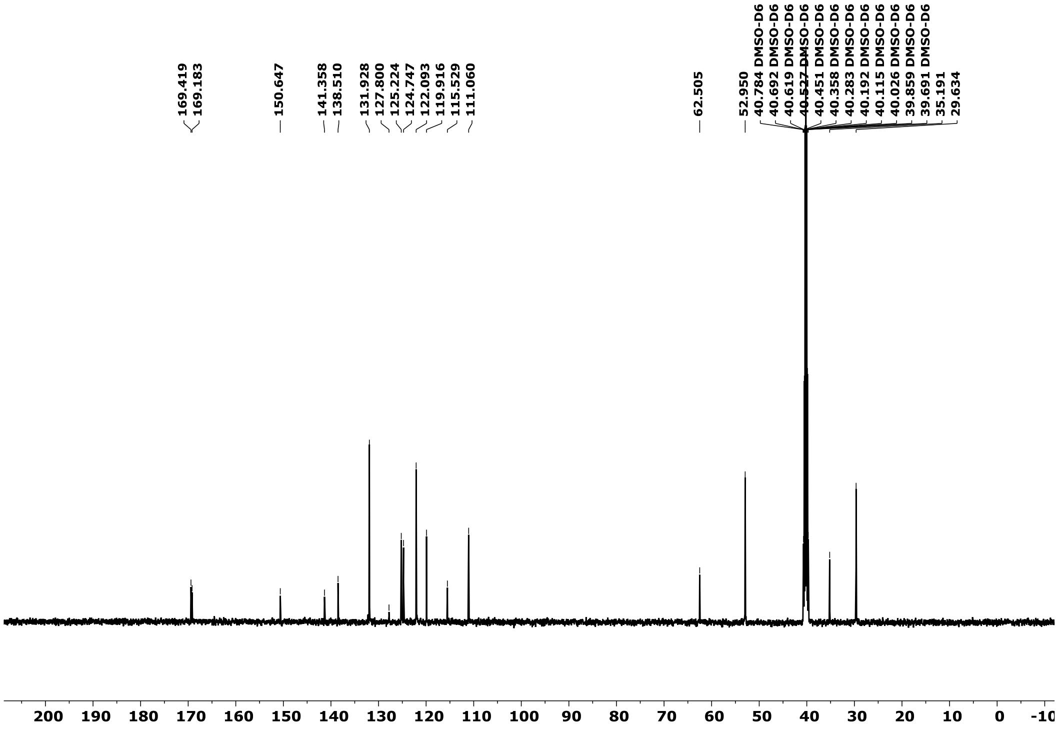

Figure S9. ^{13}C NMR (126 MHz, DMSO-d_6) spectrum of compound **5e**

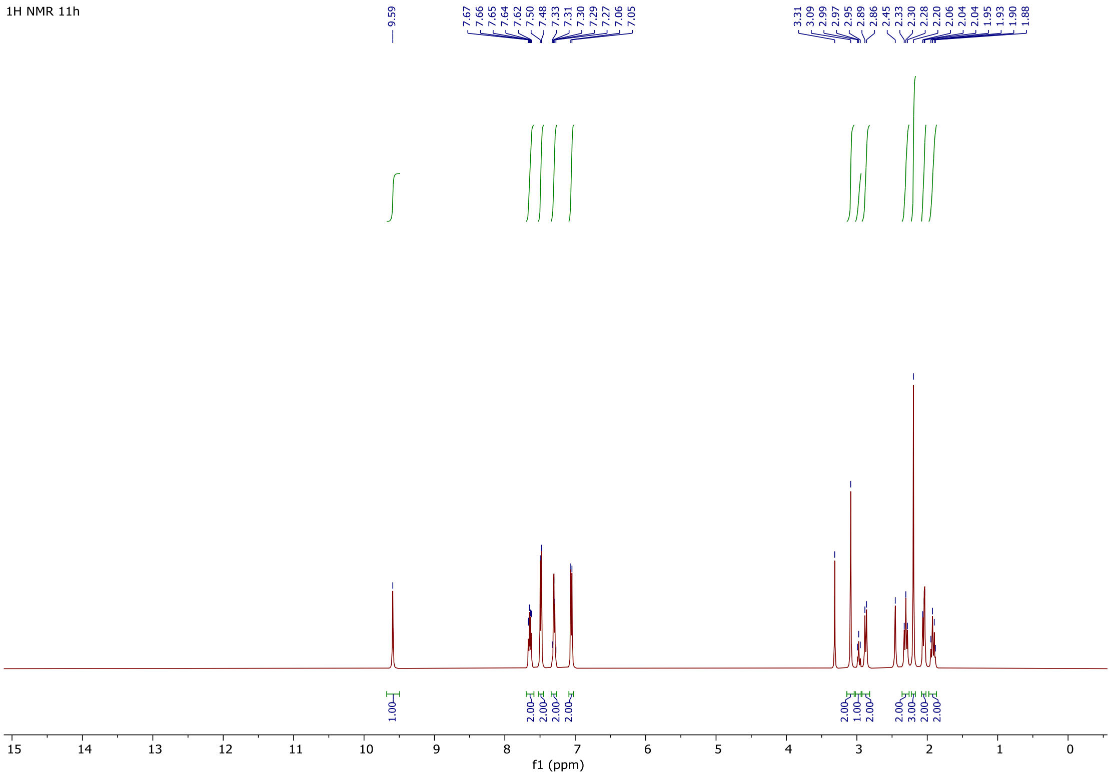

Figure S10. ^{1}H NMR (500 MHz, DMSO-d_6) spectrum of compound **5f**

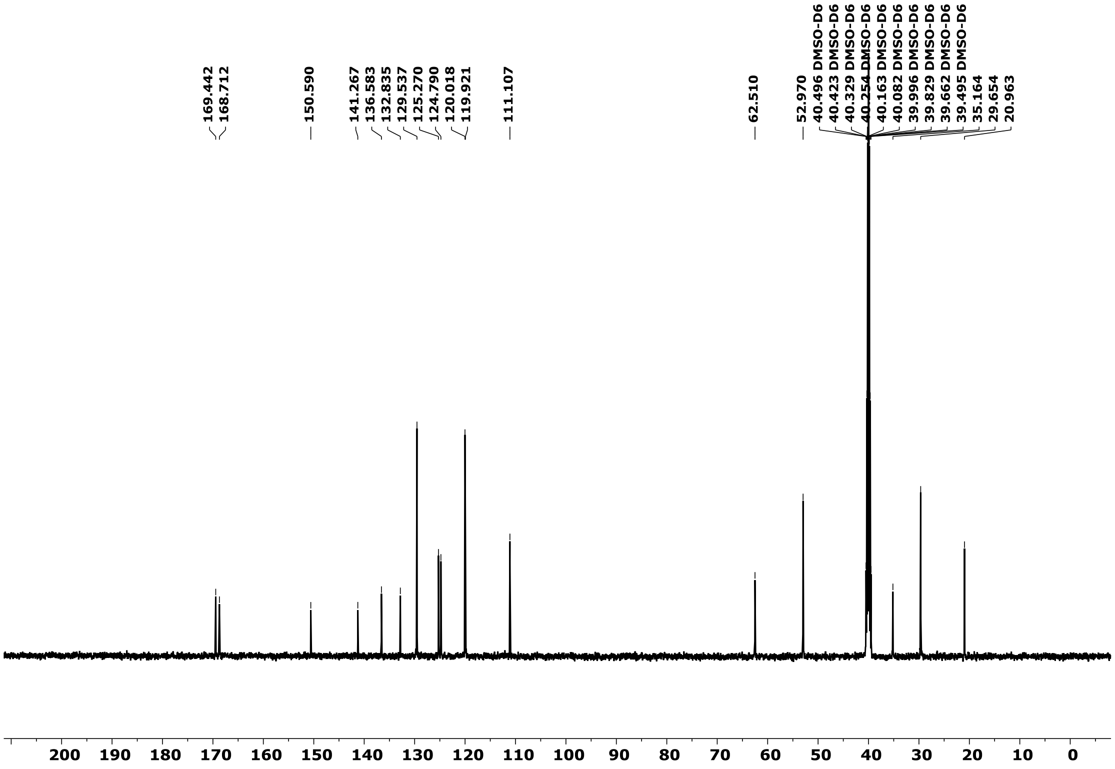

Figure S11. ^{13}C NMR (126 MHz, DMSO-d_6) spectrum of compound **5f**

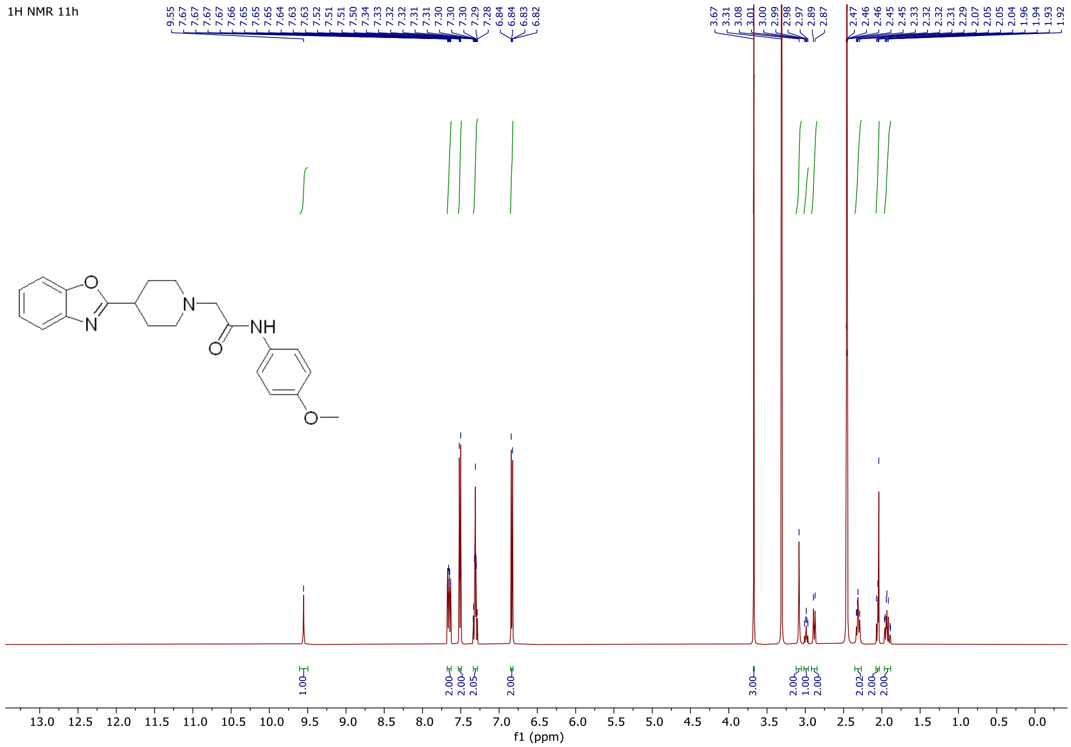

Figure S12. ^{1}H NMR (500 MHz, DMSO-d_6) spectrum of compound **5g**

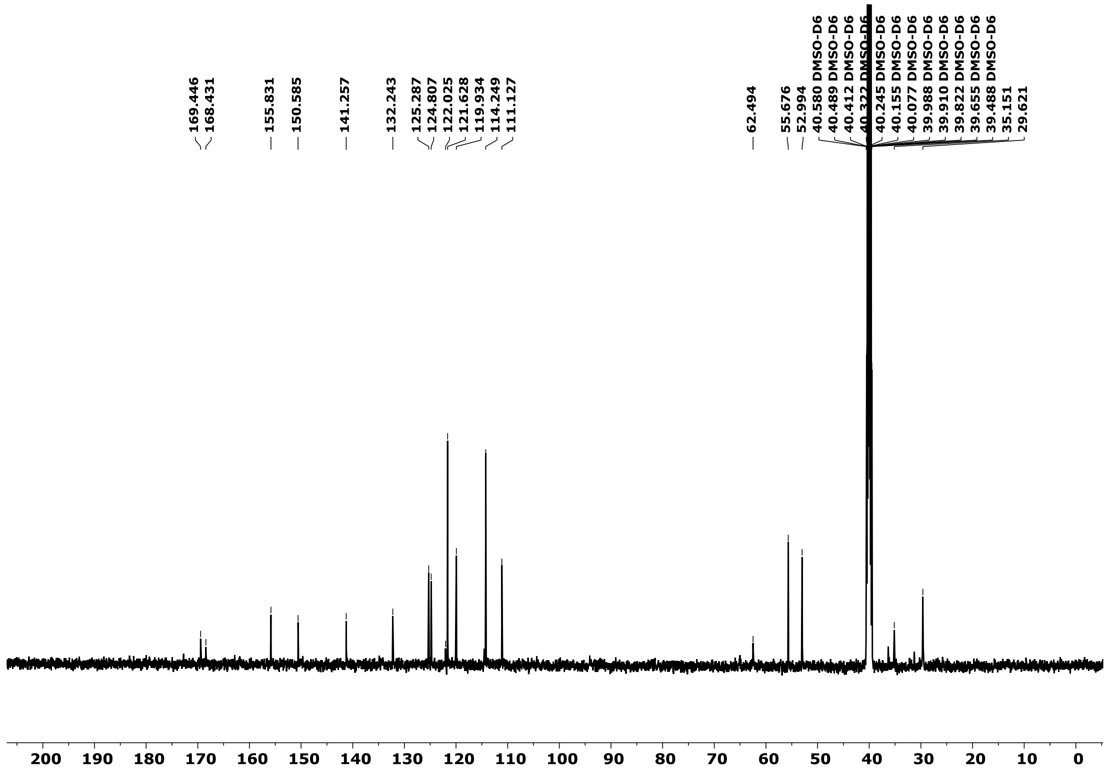

Figure S13. ^{13}C NMR (126 MHz, DMSO-d_6) spectrum of compound **5g**

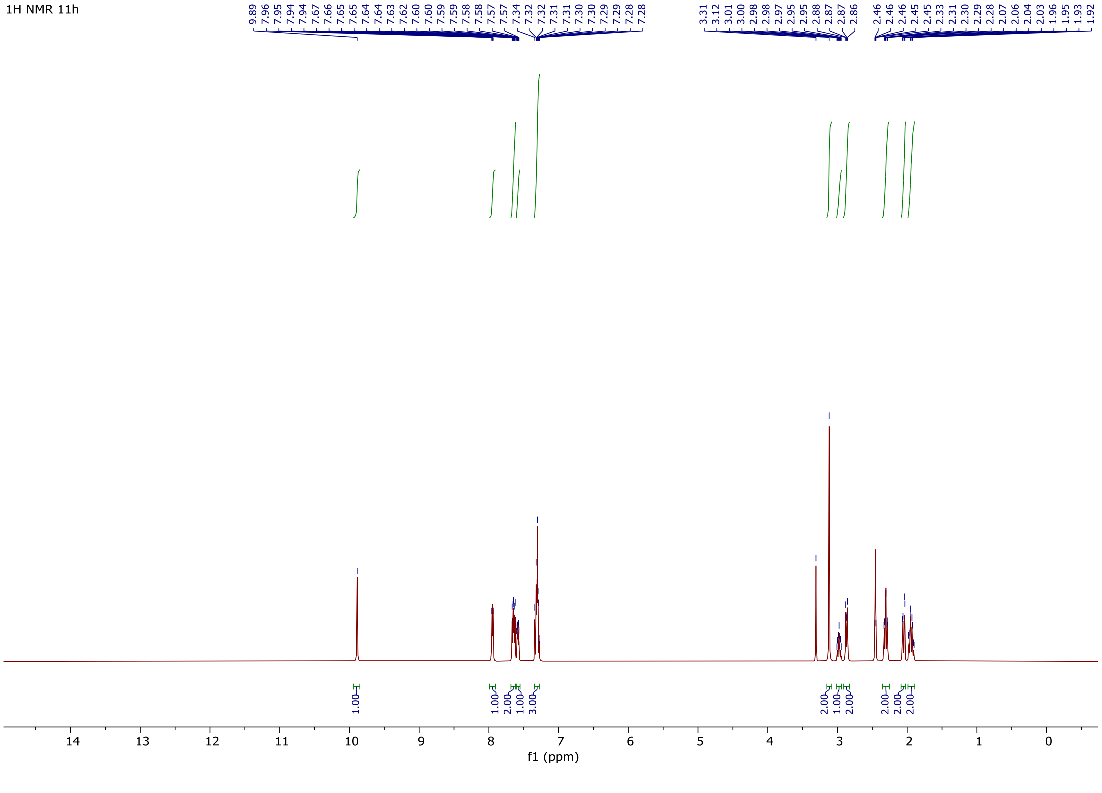

Figure S14. ^{1}H NMR (500 MHz, DMSO-d_6) spectrum of compound **5h**

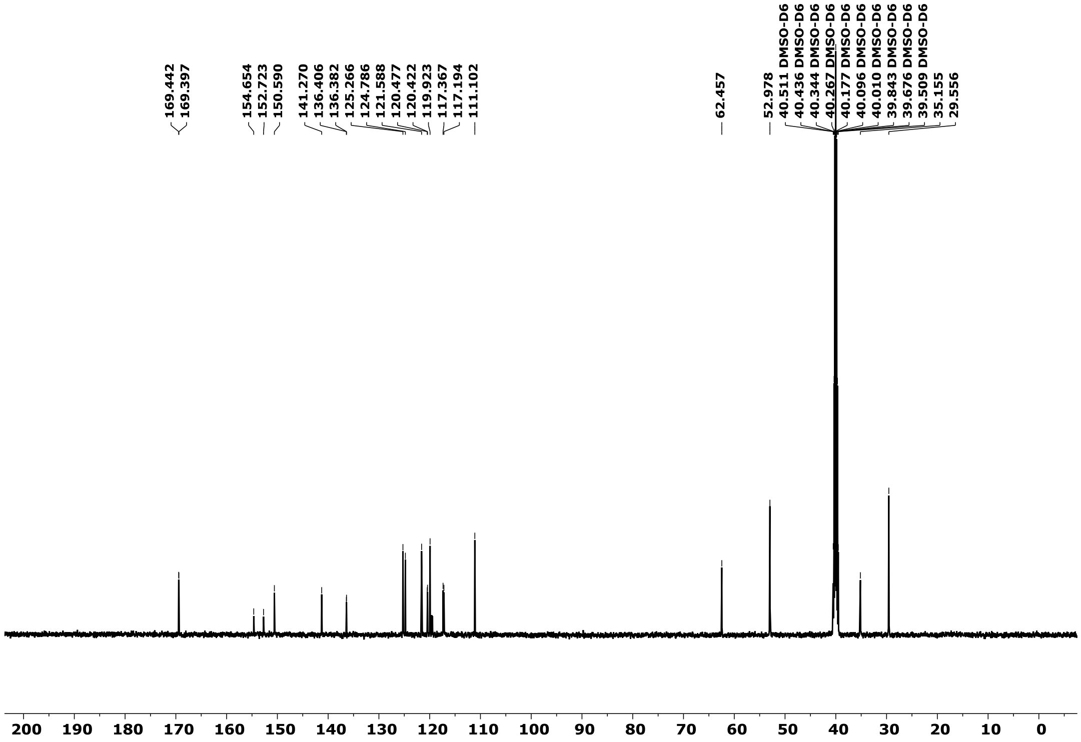

Figure S15. ^{13}C NMR (126 MHz, DMSO-d_6) spectrum of compound **5h**

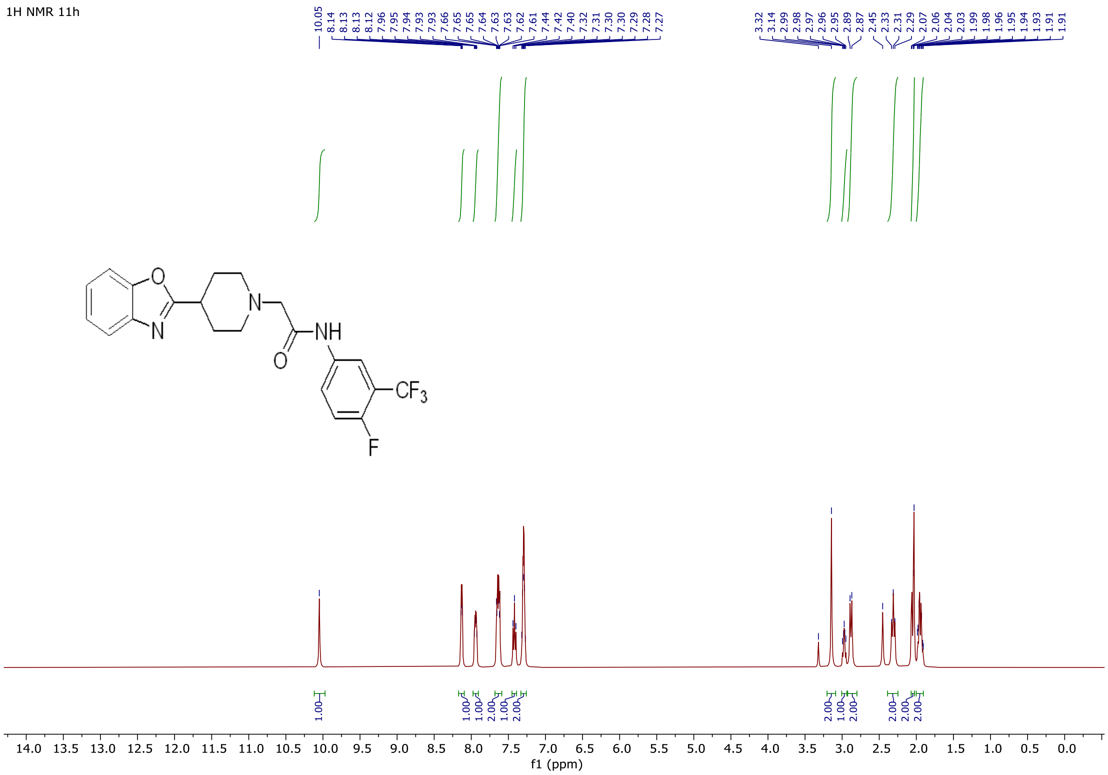

Figure S16. ^{1}H NMR (500 MHz, DMSO-d_6) spectrum of compound **5i**

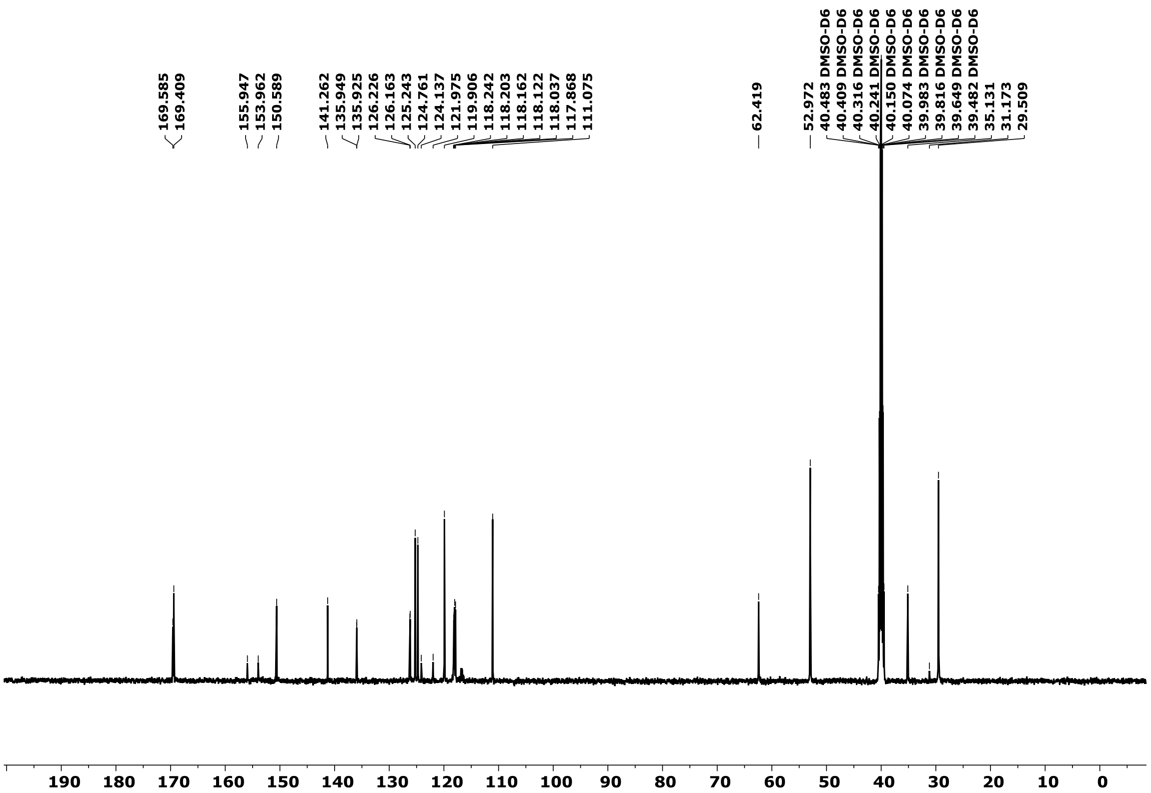

Figure S17. ^{13}C NMR (126 MHz, DMSO-d_6) spectrum of compound **5i**

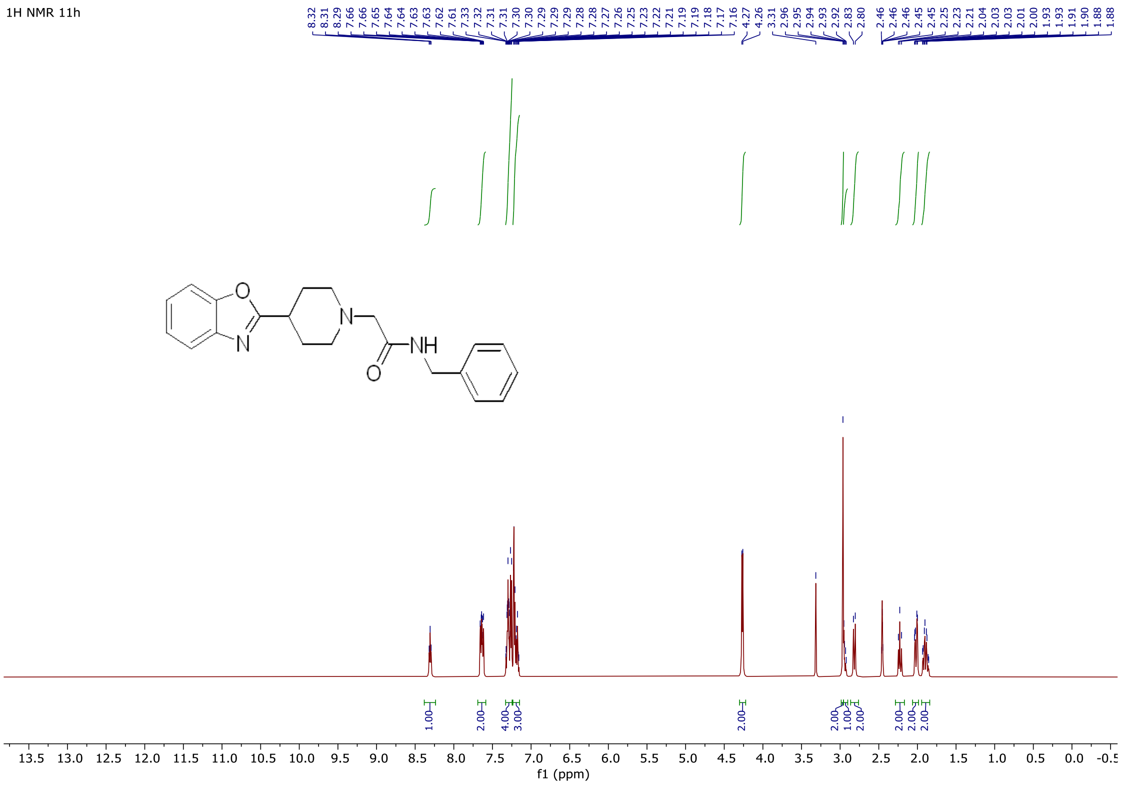

Figure S18. ^{1}H NMR (500 MHz, DMSO-d_6) spectrum of compound **8a**

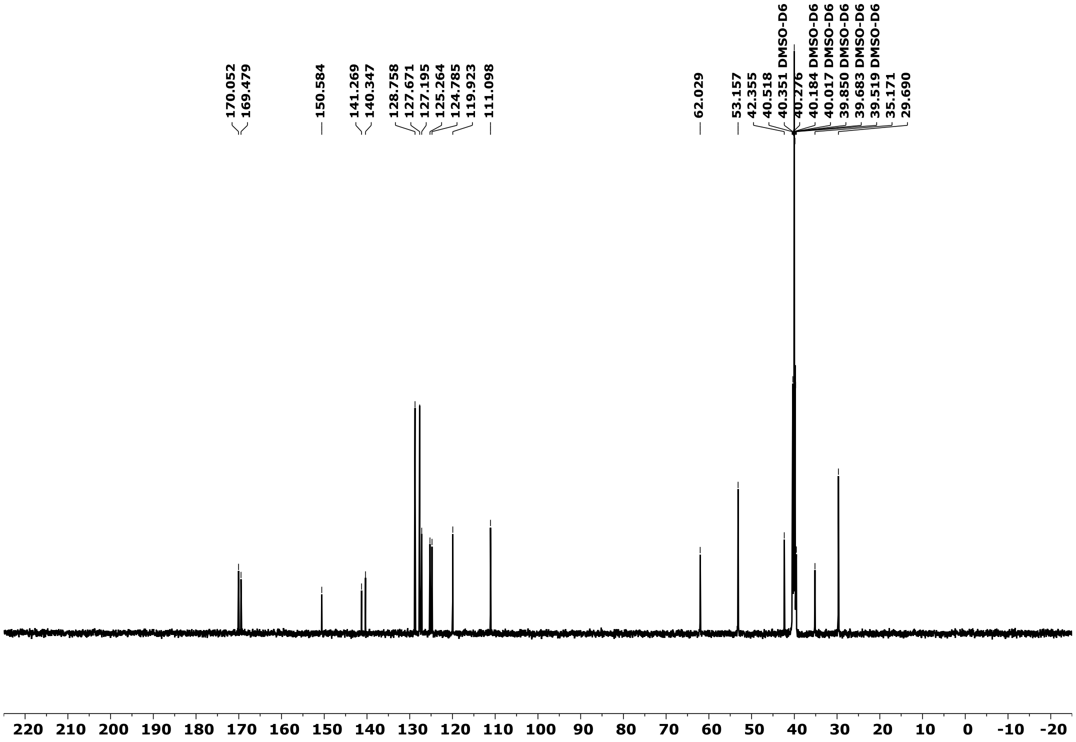

Figure S19. ^{13}C NMR (126 MHz, DMSO-d_6) spectrum of compound **8a**

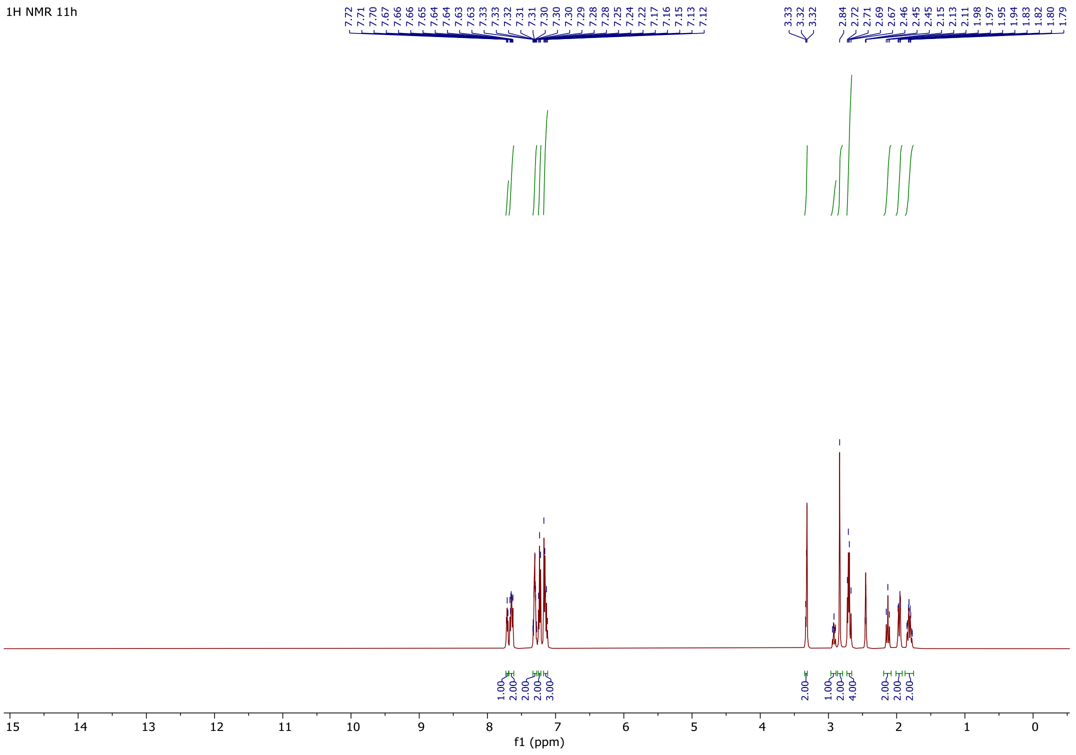

Figure S20. ^{1}H NMR (500 MHz, DMSO-d_6) spectrum of compound **8b**

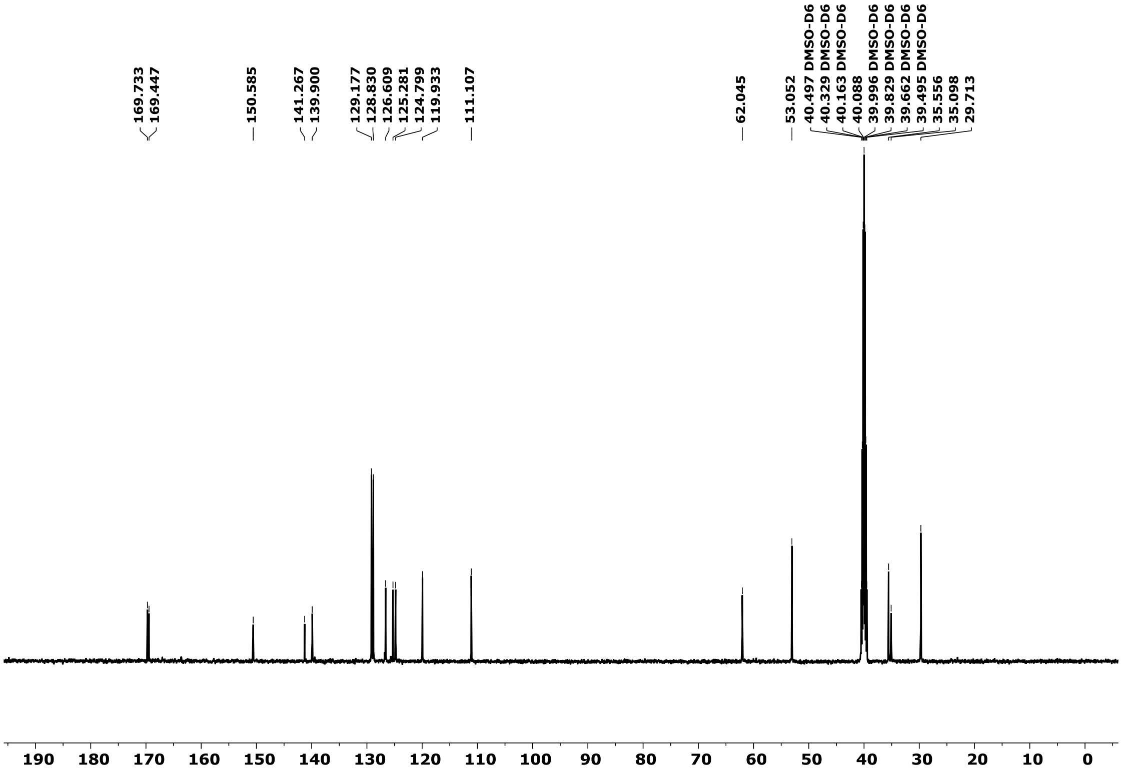

Figure S21. ^{13}C NMR (126 MHz, DMSO-d_6) spectrum of compound **8b**

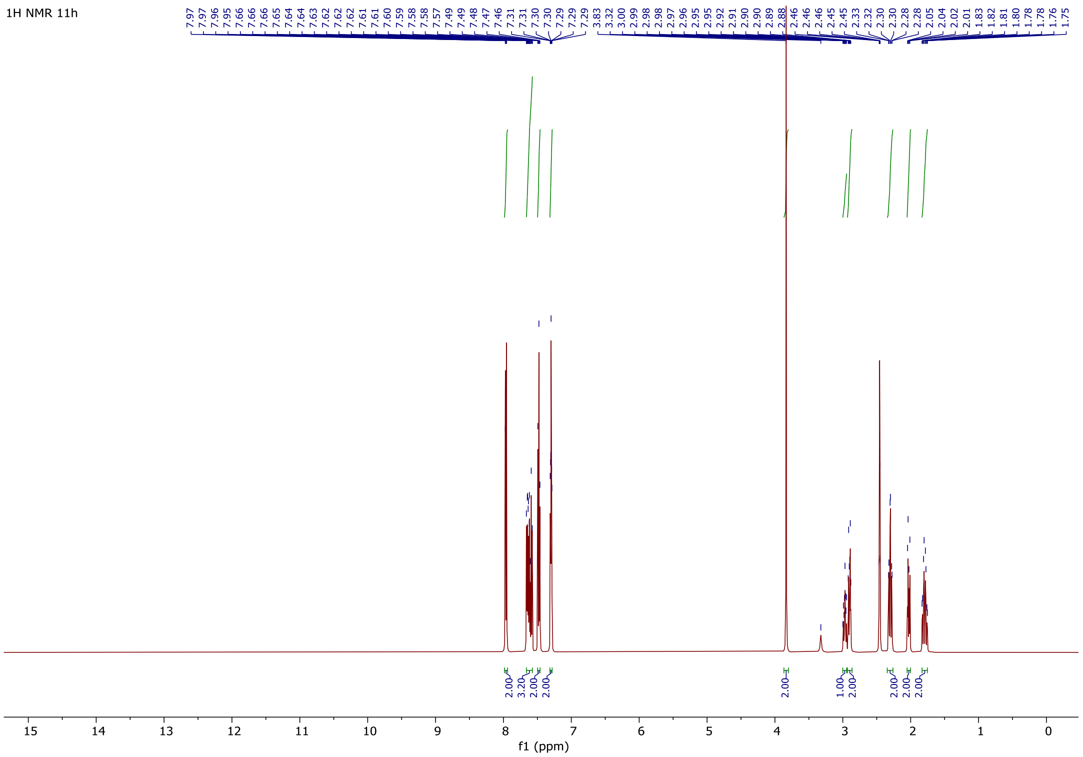

Figure S22. ^{1}H NMR (500 MHz, DMSO-d_6) spectrum of compound **11a**

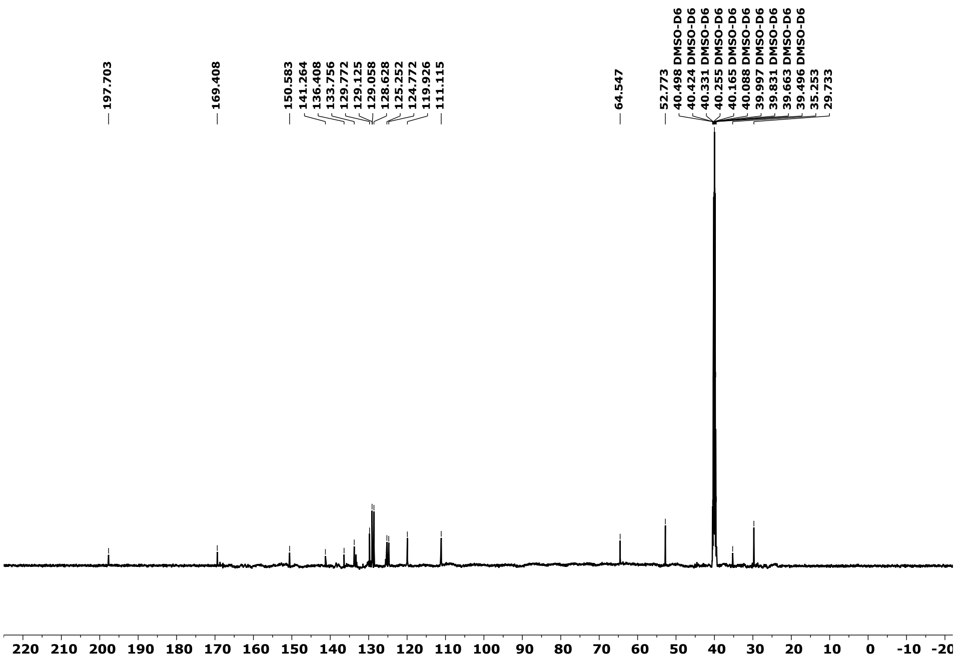

Figure S23. ^{13}C NMR (126 MHz, DMSO-d_6) spectrum of compound **11a**

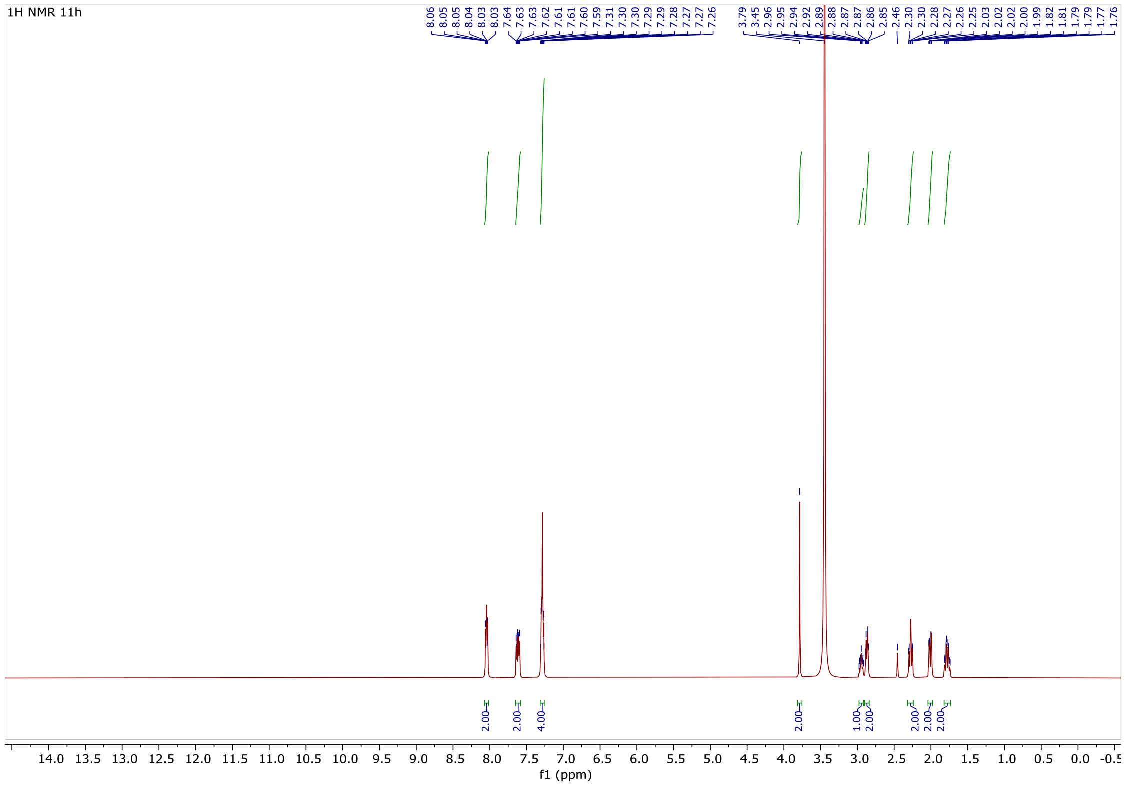

Figure S24. ^{1}H NMR (500 MHz, DMSO-d_6) spectrum of compound **11b**

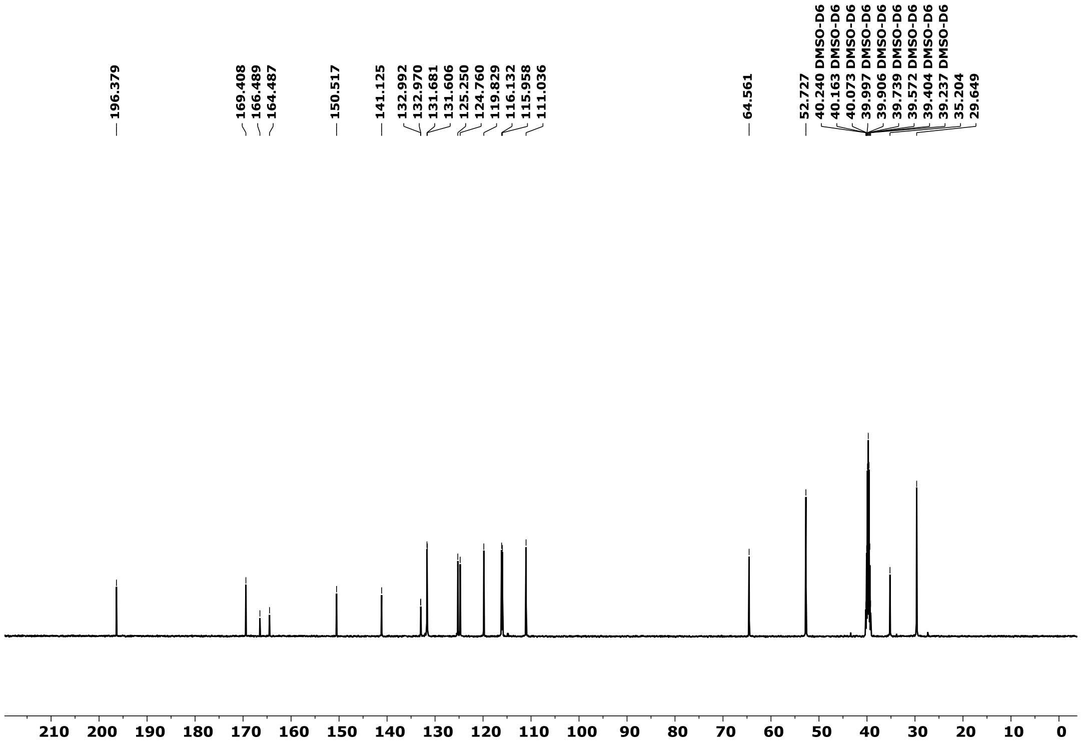

Figure S25. ^{13}C NMR (126 MHz, DMSO-d_6) spectrum of compound **11b**

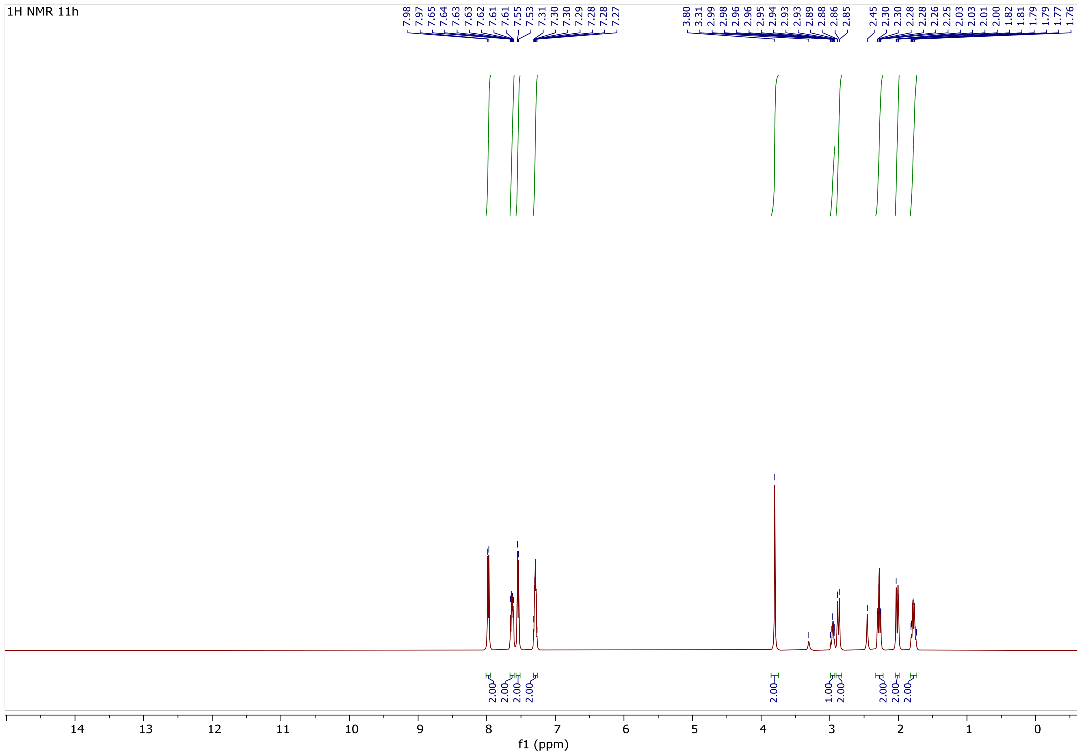

Figure S26. ^{1}H NMR (500 MHz, DMSO-d_6) spectrum of compound **11c**

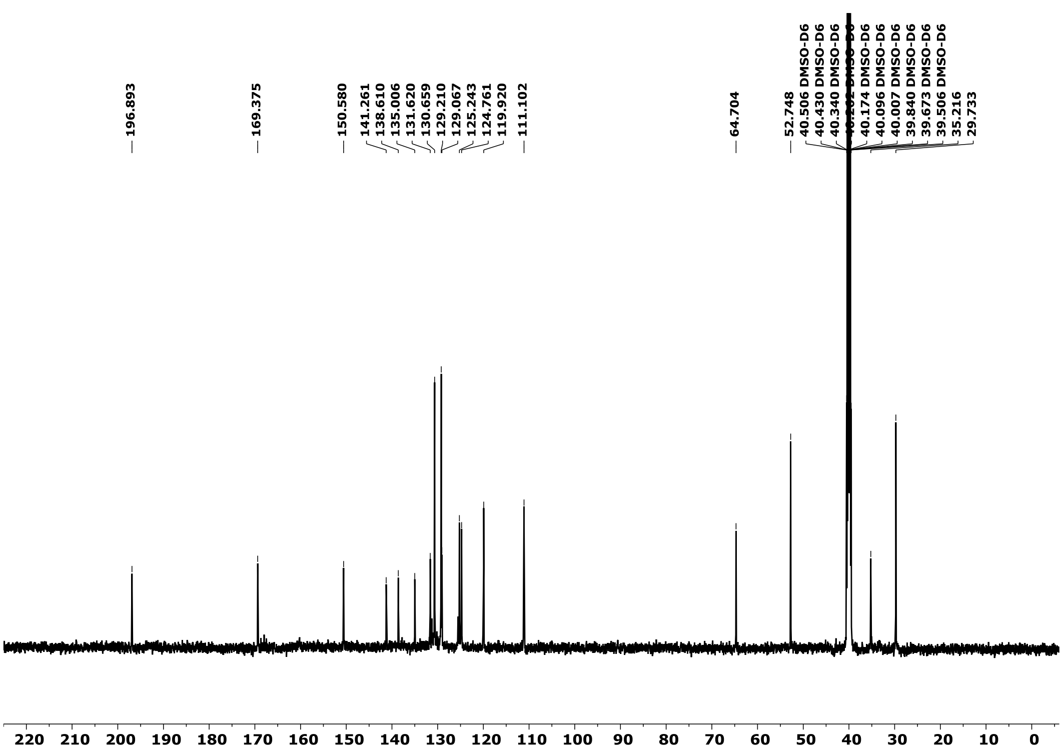

Figure S27. ^{13}C NMR (126 MHz, DMSO-d_6) spectrum of compound **11c**

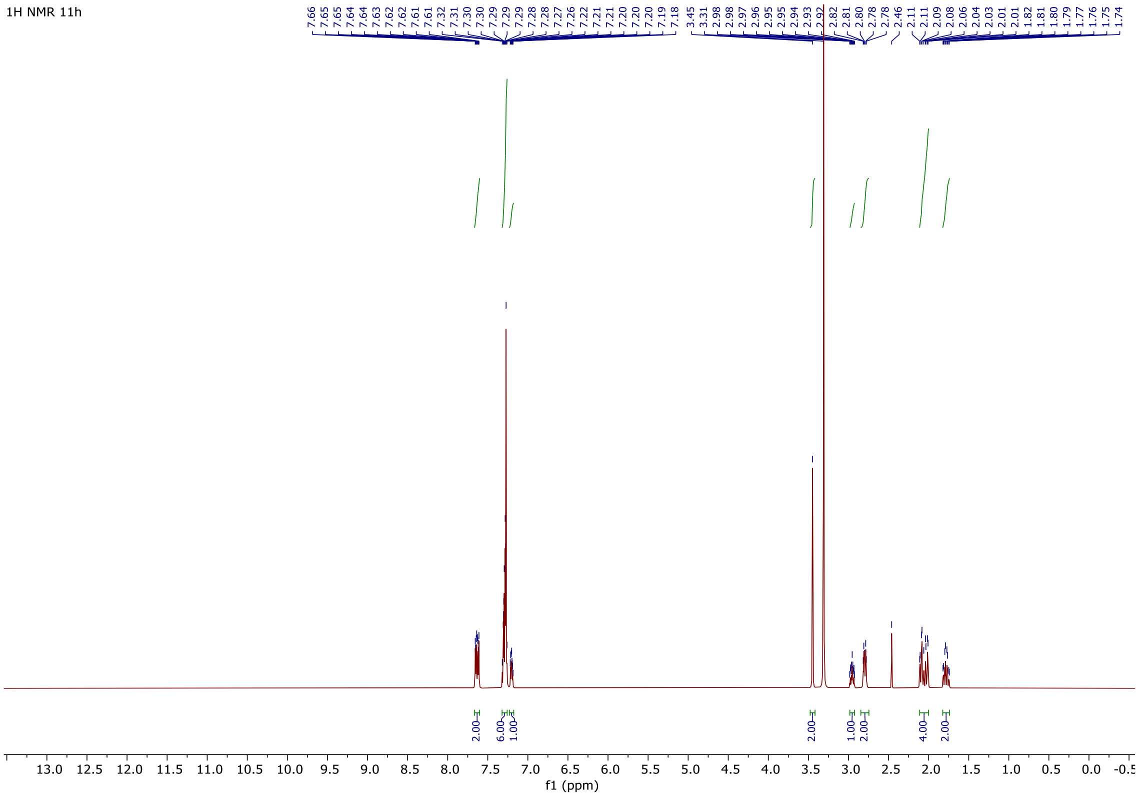

Figure S28. ^{1}H NMR (500 MHz, DMSO-d_6) spectrum of compound **13**

Table S1. The sequences for primers used in quantitative Real Time Reverse-Transcriptase PCR (qRT-PCR)

Gene	Primer sequence
**BAX**	F: 5'- CCCGAGAGGTCTTTTTCCGAG -3'<br>R: 5'- CCAGCCCATGATGGTTCTGAT -3'
**Bcl-2**	F: 5’-CCTGTG GAT GAC TGA GTA CC-3’<br>R: 5’-GAGACA GCC AGG AGA AAT CA-3’
**Caspase-9**	F: 5'- CTGAGCCAGATGCTGTCCCAT-3'<br>R: 5'- CCAAGGTCTCGATGTACCAGGAA-3'
**p53**	F: 5′-CCCCTCCTGGCCCCTGTCATCTTC-3′<br>R: 5′-GCAGCGCCTCACAACCTCCGTCAT-3′
**GAPDH**	F: 5′-GCA AGT TCA ACG GCA CGA TCA AG-3′<br>R: 5′-CTA CTC AGC ACC AGC ATC ACC-3′
